# Elevated serum polyclonal immunoglobulin free light chains in patients with severe asthma

**DOI:** 10.3389/fphar.2023.1126535

**Published:** 2023-06-16

**Authors:** Umberto Basile, Giuseppe Santini, Cecilia Napodano, Giuseppe Macis, Krizia Pocino, Francesca Gulli, Mario Malerba, Andrew Bush, Ian M. Adcock, Paolo Montuschi

**Affiliations:** ^1^ Department of Translational Medicine, Catholic University of the Sacred Heart, University Hospital Agostino Gemelli Foundation IRCCS, Rome, Italy; ^2^ Clinical Pathology Unit, Santa Maria Goretti Hospital, ASL, Latina, Italy; ^3^ Pharmacology, Faculty of Medicine, Catholic University of the Sacred Heart, Rome, Italy; ^4^ Clinical Pathology Unit, Belcolle Hospital, ASL Viterbo, Rome, Italy; ^5^ Department of Laboratory Medicine and Pathology, S. Agostino Estense Hospital, Modena, Italy; ^6^ Department of Radiological Sciences, University Hospital Agostino Gemelli Foundation IRCCS, Rome, Italy; ^7^ Clinical Pathology Unit, San Pietro Fatebenefratelli Hospital, Rome, Italy; ^8^ Clinical Biochemistry Laboratory, IRCCS, Bambino Gesù Children’s Hospital, Rome, Italy; ^9^ Respiratory Unit, S. Andrea Hospital, University of Piemonte Orientale, Vercelli, Italy; ^10^ National Heart and Lung Institute, Faculty of Medicine, Imperial College London, London, United Kingdom

**Keywords:** asthma, inflammation, free light chains, biomarkers, personalised medicine

## Abstract

**Background:** Inflammation plays a pivotal role in the pathophysiology of asthma. Free light chains (FLC) can cause inflammation by mast cell antigen-activation. Serum immunoglobulin (Ig) FLC κ, but not λ, were shown elevated in adult males with asthma. We sought to investigate if serum Ig FLC concentrations are affected by asthma severity and their relationships with inflammatory outcomes.

**Methods:** By using immunoassays, we measured serum κ and λ Ig FLCs in 24 severe persistent asthma patients, 15 patients with moderate persistent asthma, 15 steroid-naïve mild persistent asthma patients and 20 healthy control subjects in a cross-sectional observational study. Total and specific serum IgE concentrations, fractional exhaled nitric oxide (F_E_NO), lung function, peripheral blood eosinophils and neutrophils, and C reactive protein (CRP) were also measured.

**Results:** Serum κ FLC concentrations were elevated in severe asthma patients compared mild asthma patients (*p* < 0.05) and healthy subjects (*p* < 0.05). Serum λ FLCs were higher in severe asthma patients than in healthy subjects (*p* < 0.05) and correlated with blood eosinophil counts (percentage, κ: *r* = 0.51, *p* = 2.9678^−6^; λ: *r* = 0.42, *p* = 1.7377^−4^; absolute values, κ: *r* = 0.45, *p* = 6.1284^−5^; λ: *r* = 0.38, *p* = 7.8261^−4^), but not with total or specific serum IgE. In severe asthma patients, serum Ig FLC correlated with serum CRP (κ: *r* = 0.33; *p* = 0.003; λ: *r* = 0.38, *p* = 8.8305^−4^) and blood neutrophil cell counts (percentage, κ: *r* = 0.31; *p* = 0.008; λ: *r* = 0.29, *p* = 0.01; absolute values, κ: *r* = 0.40; *p* = 3.9176^−4^; λ: *r* = 0.40, *p* = 4.5479^−4^), were elevated in subjects with blood eosinophilia (≥300 cells/µL) (*n* = 13) compared with non-eosinophilic subjects (*n* = 10) (κ: 19.2 ± 1.2 mg/L *versus* 12.1 ± 1.3 mg/L, *p* < 0.001; λ: 27.2 ± 2.6 mg/L *versus* 16.8 ± 2.5 mg/L, *p* < 0.01), but were similar in atopic (*n* = 15) *versus* nonatopic subjects (*n* = 9) (κ: *p* = 0.20; λ: *p* = 0.80). Serum FLC were negatively correlated with lung function tests, including forced expiratory volume in one second (FEV1) (κ: *r* = −0.33; *p* = 0.0034; λ: *r* = −0.33; *p* = 0.0035), and FEV_1_/forced vital capacity ratio (κ: *r* = −0.33; *p* = 0.0034; λ: *r* = −0.33; *p* = 0.0036).

**Conclusion:** Serum Ig FLCs are elevated in severe asthma adults and might represent new surrogate markers of inflammation. The pathophysiological implications of these findings require further research. This study was approved by the ethics committee of the University Hospital Agostino Gemelli Foundation and Catholic University of the Sacred Heart (approval number P/1034/CE2012).

## Introduction

Asthma is a heterogeneous disease characterized by chronic airway inflammation and recurrent episodes of bronchospasm (Global Initiative for Asthma, 2023). Several inflammatory and immune cells, including mast cells, dendritic cells, eosinophils, T cells and neutrophils are involved in the pathophysiology of asthma (Dudeck et al., 2011; Mandlik and Mandlik, 2020). In particular, mast cells contribute to asthma airway inflammatory processes through immunoglobulin (Ig) E-dependent or non-IgE-dependent mechanisms (Galli, and Costa, 1995; Galli, 1997; Galli and Tsai, 2012; Bradding and Saito, 2019; Galli et al., 2020). Mast cells are elevated in bronchial biopsies in both atopic and nonatopic adults with asthma and airway submucosal glands in asthma, and the extent of degranulation of these cells is directly related to disease severity (Amin, et al., 2000; Carroll et al., 2002).

Polyclonal immunoglobulin free light chains (FLC) were found elevated in various biological fluids in a number of chronic inflammatory diseases (Brebner and Stockley, 2013). Urinary FLC concentrations, which correlate with the degree of systemic inflammation, are elevated in patients with chronic inflammatory rheumatic disease (Bramlage et al., 2016); serum FLC concentrations, correlating with disease activity, were found elevated in patients with systemic lupus erythematosus (Draborg et al., 2015). One study showed elevated serum κ, but not λ, Ig FLCs in both atopic and nonatopic asthma patients compared with atopic and nonatopic healthy control subjects (Kraneveld et al., 2005). However, only men were included in this study and no information on asthma severity was provided (Kraneveld et al., 2005). FLC are active ubiquitous molecules that are able to trigger and/or participate in inflammatory processes through the antigen-specific activation of mast cells (Napodano et al., 2019). FLC can also exert other biological activities such as the ability to bind intracellular and extracellular proteins, enzymatic activity and the modulation of cellular interactions (Sun et al., 1994; Paul et al., 1995; Dispenzieri et al., 2009; Hutchison and Landgren, 2011; Mortaz et al., 2017).

One of the major complications in the pathophysiology of asthma is airway remodeling (Jeffery, 2001; Global Initiative for Asthma, 2023). Identification of non-invasive biomarkers of airway inflammation and remodelling is a priority in asthma research (Montuschi and Barnes, 2011).

To date, the “gold standard” for evaluation of airway remodeling is bronchoscopy and bronchial biopsy. These methods are invasive and not used as a routine in persons with asthma, who have a higher risk of bronchospasm due to airway hyper-responsiveness (Global Initiative for Asthma, 2023).

Discovery of new non-invasive immunological markers of asthma has a potential clinical utility for diagnosis, assessment and monitoring of this disease. The objectives of our study were to investigate if serum Ig FLC concentrations are affected by asthma severity and their relationships with inflammatory outcomes.

## Methods

### Study design

This was a single center, prospective, proof of concept observational cross-sectional study including one visit. Participants attended the Clinical Pharmacology Unit clinic, Catholic University of the Sacred Heart, University Hospital Agostino Gemelli Foundation IRCCS, Roma, Italy, on one occasion for spirometry, fractional exhaled nitric oxide (*F*_E_NO) measurement, and blood sampling for blood cell count test and measurement of serum total and specific IgE and Ig FLC concentrations. Peripheral blood neutrophil counts and serum C reactive protein (CRP) were measured in persons with severe asthma.

This study was conducted in compliance with the 1975 Declaration of Helsinki, revised in 2004, and approved by the ethics committee of the University Hospital Agostino Gemelli Foundation and Catholic University of the Sacred Heart (approval number P/1034/CE2012). Written consent was obtained from participants.

### Study subjects

This study included 54 adults with asthma (24 with severe persistent asthma, 15 with moderate persistent asthma and 15 with mild persistent asthma) and a control group consisting of 20 healthy subjects. Diagnostic criteria for asthma and classification of asthma have been reported elsewhere (Shaw et al., 2015). Asthma severity was defined by the level of pharmacotherapy required for asthma control (Chung et al., 2014; Global Initiative for Asthma, 2023). Persons with severe persistent asthma were being treated with high dose inhaled corticosteroids (ICS) plus long-acting β_2_-agonists (LABA) and/or oral corticosteroids; persons with moderate persistent asthma were on maintenance therapy with low to medium dose of ICS plus LABA; persons with mild persistent asthma were steroid-naïve. Ex-smokers had a smoking history > 5 pack-years. Atopy was assessed by measuring serum specific IgE against a panel of common aeroallergens. Subjects with asthma having serum specific IgE concentrations > 1 kU/L against the aeroallergen tested were considered atopic. Participants had no respiratory tract infections in the previous 3 weeks.

### Pulmonary function testing

Spirometry was performed with a Pony FX spirometer (Cosmed, Rome, Italy) according to standardised international procedures (Miller et al., 2005). At least three acceptable maneuvers were performed with the two best ones having a maximal difference in FEV_1_ and forced vital capacity (FVC) of 150 mL. The best of three consecutive maneuvers was chosen. To perform the bronchodilator reversibility test, participants were asked to perform a spirometry, inhale 400 μg of salbutamol and repeat the spirometry after 10 min.

### F_E_NO measurement

We measured F_E_NO using an electrochemical sensor F_E_NO analyser (NIOX MINO^®^, Aerocrine, Stockholm, Sweden) (Menzies et al., 2007). F_E_NO was measured on-line using the single breath method at constant flow of 50 mL/s according to standardised procedures (American Thoracic Society and European Respiratory Society, 2005; Dweik et al., 2011). Measurement of F_E_NO was obtained before spirometry.

### Blood sampling

A whole blood sample was obtained from participants in the study for measurement of serum Ig FLC, serum total and specific IgE concentrations, and blood cell count test. Serum CRP concentrations were measured in severe asthma participants. Blood samples were centrifuged at 4,000 g for 5 min at room temperature and serum aliquots stored at −80°C until the assays were performed.

### Measurement of serum Ig FLC

The nephelometric measurement of FLC was performed using monoclonal antibodies in the N Latex κ and λ FLC kit (Siemens Healthcare Diagnostics Products GmbH, Marburg, Germany). FLC in the serum samples were assayed in a batch mode on the BNTMII automatic analyzer (Siemens Healthcare Diagnostics Ltd., Erlangen, Germany) according to the instructions provided by the manufacturer.

This method showed high precision, freedom from a high-dose hook effect and good lot-to-lot consistency (Velthuis et al., 2011). Reproducibility of serum FLC measurements ranges from 4% to 7% (Velthuis et al., 2011). Comparisons with Freelite™, an independent analytical method, showed concordance rates for serum κ FLC, λ FLC and κ/λ FLC ratio of 99.2%, 94.2%, and 95%, respectively (Velthuis et al., 2011).

### Measurement of total and specific serum IgE

Serum total IgE were measured using ImmunoCAP Total IgE applied on Phadia instrumentation (Thermo Scientific, Uppsala Sweden). Following the Clinical and Laboratory Standards Institute (CLSI) EP28-A3C guidelines (Clinical and Laboratory Standards Institute, 2010), we tested 20 healthy blood donors from the local population and set serum total IgE ≥ 100 lU/L as cut-off positive.

Mesaurement of specific IgE in serum was carried out with the Immulite^®^ 2000 AlaTOP Allergy Screen chemiluminescence immunoassay (Siemens Medical Solutions-Diagnostics, New Jersey, United States) performed according to the manufacturer instructions.

AlaTOP represents a screening kit for the major inhalant allergens: Dermatophagoides pteronyssinus, cat dander, dog dander, Cynodon dactylon, Phleum pretense L., birtch, Cryptomerica japonica, Penicillium notatum, Alternaria tenuis, Ambrosia artemisifolia L., Plantago lanceolata L., and Parietaria officinalis.

Qualitative results were expressed as signal/cut-off ratio (s/co) and deemed positive if s/co ≥ 1.1 or negative if s/co < 1.1 as follows:

$$\frac{s}{co}=\frac{cps\;sample\;or\;control}{cps\;Calibrator\;x\;P1}$$

cps = average count per second
P1 = parameter 1 of the standard curve
s/co = signal/cut-off ratio.

### Statistical analysis

There are no data in the literature to inform a power calculation, so the sample size was opportunistic. Statistical analysis was performed using GraphPad Prism 6.0 and MetaboAnalyst 5.0. For continuous data, values are expressed as mean ± SEM or median and interquartile range (25th and 75th percentiles), after assessing for normality with D'Agostino-Pearson omnibus normality test. Depending on data distribution, ANOVA or Kruskal-Wallis test was used for between group comparisons and overall *p* values are shown. To reduce the chance of type I error, *post hoc* Tukey’s multiple comparisons test or Fisher’s LSD *post hoc* test (MetaboAnalyst 5.0 available at https://www.metaboanalyst.ca/) or *post hoc* Dunn’s multiple comparisons test was used and *post hoc* test *p* values are reported. Adjusted *p* values were calculated by false discovery rate (FDR) at a cut-off value of 0.05. Categorical data are expressed as numbers. Fisher’s exact test was used for comparing two groups; Chi square test was used for comparing more than two groups. Significance was defined as a value of *p* < 0.05. The correlation matrix and relative *p* values used for generating the heatmap are provided in the Supplementary Material (correlation table.xlsx and *p* values correlation table.xlsx files).

We used the Debiased Sparse Partial Correlation (DSPC) network (Basu et al., 2017) to visualise the correlation network. The nodes are input variables, while the edges represent the association measures. For better visualization, the default DSPC network only shows the top correlations (edges) based on their *p*-value rankings (top 20% when the total number of edges is less than 1000).

Due to the exploratory nature of the study, a formal sample size calculation was not required.

## Results

**FIGURE 1:**
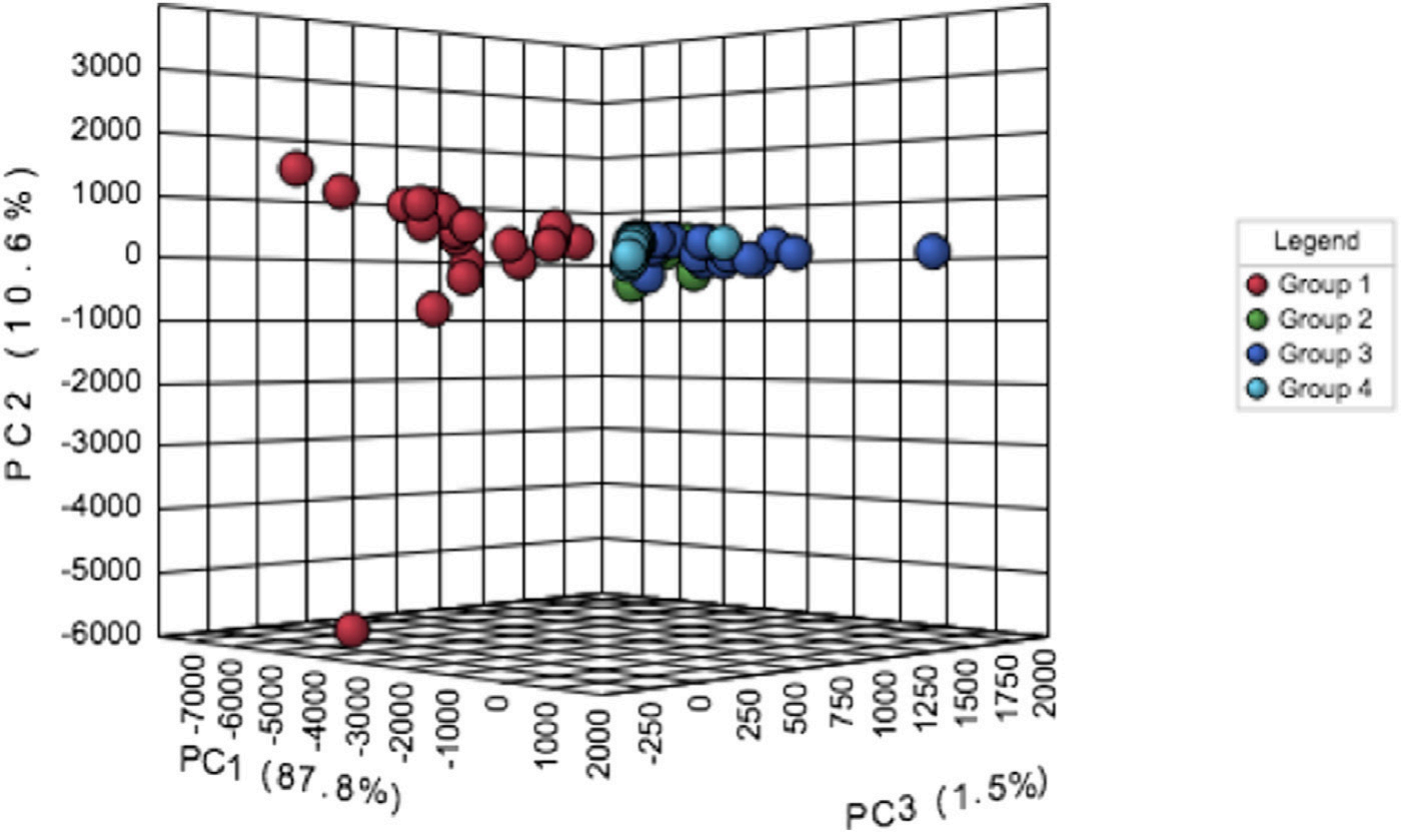
Principal component analysis (PCA) of data related to persistent severe asthma (group 1, red dots), persistent moderate asthma (group 2, green dots), persistent mild asthma (group 3, blue dots), and healthy control participants (group 4, light blue dots).

Principal component analysis (PCA) shows that features of the severe persistent asthma group were strikingly different from those observed in the other study groups (Figure 1). This huge difference was responsible for the lack of separation of mild persistent asthma, moderate persistent asthma and healthy control groups (Figure 1). PCA also shows variability within severe asthma group (Figure 1).

### Study subjects

A total of 74 persons 39 women and 35 men, aged 18–82 years, participated in this study, including 24 adults with persistent severe asthma, 15 adults with persistent moderate asthma, 15 adults with persistent mild asthma, and 20 healthy control adults (Table 1). Participants with severe persistent asthma were on maintenance treatment with inhaled fluticasone propionate > 500 μg/day combined with a LABA and/or oral corticosteroids; participants with moderate persistent asthma were on maintenance treatment with inhaled fluticasone propionate ≤ 500 μg/day, 13 out of 15 in combination with a LABA; participants with mild persistent asthma were steroid naive or not being received ICS for at least 3 months.

**TABLE 1:** Subject characteristics^a^.

	Severe asthma subjects (*n* = 24)	Moderate asthma subjects on ICS (*n* = 15)	Steroid-naive mild asthma subjects (*n* = 15)	Healthy control subjects (*n* = 20)	^b^ *p* values
Age, years	62.5 (55.0–69.8)^1^ ^,^ ^2^	46.0 (33.0–66.0)	32.0 (29.0–39.0)^1^	33.0 (28.0–53.0)^2^	<0.0001
Gender, F/M	15/9	7/8	6/9	11/9	0.54
^c^Atopy yes/no	15/9^3^	10/5^4^	15/0^3^ ^,^ ^4^ ^,^ ^5^	9/11^5^	0.008
^d^Current smokers/ex-smokers/non-smokers	3/5/16	1/6/8	5/0/10	6/0/14	0.76
Smoking history, pack/years	0 (0–18.2)	0 (0–12.8)	0 (0–1.3)	0 (0–1.8)	0.53
^e^ICS, yes/no	21/3	15/0	0/15	0/20	0.51
^f^Oral corticosteroids, yes/no	6/18	0/15	0/15	0/20	n.a
^g^Blood eosinophils, absolute cell counts (cells/μL)	381^6^ (126.0–639.0)	180.0 (97.0–260.0)	230.0 (145.0–265.0)	121.5^6^ (91.5–225.0)	<0.05
^g^Blood eosinophils, % cell counts	5.6 (2.1–7.3)^7^	2.8 (1.4–4.6)	3.4 (2.2–4.3)	2.1 (1.0–3.1)^7^	<0.05
^g^Blood neutrophils, absolute cell counts (cells/μL)	4,565 (3,980–5,320)	3,990 (3,210–5,350)	3,030 (2,750–4,160)	4,250 (2,571–4,477)	n.s
^g^Blood neutrophils, % cell counts	57 (54.1–61.2)	56-8 (54.4–68.1)	55.1 (47.3–60.2)	60.9 (53.4–66.0)	n.s
^g^Blood lymphocytes, absolute cell counts (cells/μL)	2,198 ± 156	1936 ± 176	2,319 ± 183	1855 ± 99	n.s
^g^Blood lymphocytes, % cell counts	28.4 (25.2–31.3)	30.3 (22.2–34.1)	36.0 (29.7–41.2)	30.9 (26.1–36.7)	n.s
^g^Blood monocytes, absolute cell counts (cells/μL)	592 ± 11^8^ ^,^ ^9^ ^,^ ^10^	420 ± 39^8^	372 ± 22^9^	341 ± 26^10^	<0.0001
^g^Blood monocytes, % cell counts	7.4 ± 0.5^11^	6.2 ± 0.4	6.0 ± 0.4	5.3 ± 0.3^11^	<0.01
Pre-BD FEV_1_, L	1.4 ± 0.7^12^ ^,^ ^13^ ^,^ ^14^	2.8 ± 0.3^12^ ^,^ ^15^	3.7 ± 0.2^13^ ^,^ ^15^	3.6 ± 0.2^14^	<0.0001
Pre-BD FEV_1_, % predicted value	57.1 ± 3.9^16^ ^,^ ^17^ ^,^ ^18^	89.6 ± 5.0^16^	99.0 ± 3.8^17^	103.5 ± 3.2^18^	<0.0001
Pre-BD FVC, L	2.4 ± 0.2^19^ ^,^ ^20^ ^,^ ^21^	3.8 ± 0.4^19^	4.6 ± 0.3^20^	4.4 ± 0.3^21^	<0.0001
Pre-BD FVC, % predicted value	80.4 ± 4.0^22^ ^,^ ^23^ ^,^ ^24^	102 ± 5.6^22^	101.8 ± 3.6^23^	104.1 ± 3.2^24^	<0.0001
Pre-BD PEF, L/s	3.9 ± 0.5^25^ ^,^ ^26^ ^,^ ^27^	7.4 ± 0.7^25^	9.1 ± 0.6^26^	8.6 ± 0.6^27^	<0.0001
Pre-BD PEF, % predicted value	58.4 ± 5.8^28^ ^,^ ^29^ ^,^ ^30^	96.7 ± 5.9^28^	108.2 ± 5.1^29^	106.7 ± 4.3^30^	<0.0001
Pre-BD FEV_1_/FVC, %	57.6 ± 2.1^31^ ^,^ ^32^ ^,^ ^33^	73.2 ± 2.5^31^ ^,^ ^34^ ^,^ ^35^	81.9 ± 1.7^32^ ^,^ ^34^	84.3 ± 1.0^33^ ^,^ ^35^	<0.0001
Pre-BD FEF_25%–75%_, L/s	0.8 ± 0.1^36^ ^,^ ^37^ ^,^ ^38^	2.2 ± 0.3^36^ ^,^ ^39^ ^,^ ^40^	4.0 ± 0.3^37^ ^,^ ^39^	3.8 ± 0.2^38^ ^,^ ^40^	<0.0001
Pre-BD FEF_25%–75%_, % predicted value	24.4 ± 2.7^41^ ^,^ ^42^ ^,^ ^43^	53.7 ± 6.1^41^ ^,^ ^44^ ^,^ ^45^	86.2 ± 5.0^42^ ^,^ ^44^	94.1 ± 4.4^43^ ^,^ ^45^	<0.0001
Post-BD FEV_1_, L	1.7 ± 0.2^46^ ^,^ ^47^ ^,^ ^48^	3.1 ± 0.3^46^	3.9 ± 0.2^47^	3.7 ± 0.2^48^	<0.0001
Post-BD FEV_1_, % predicted value	69.4 ± 3.9^49^ ^,^ ^50^ ^,^ ^51^	97.9 ± 5.7^49^	103.4 ± 3.8^50^	106.3 ± 3.1^51^	<0.0001
Post-BD FVC, L	2.7 ± 0.2^52^ ^,^ ^53^ ^,^ ^54^	3.9 ± 0.4^52^	4.6 ± 0.3^53^	4.2 ± 0.3^54^	<0.0001
Post-BD FVC, % predicted value	91.3 ± 3.6	103.8 ± 5.3	102.6 ± 3.1	103.3 ± 3.0	<0.05
Post-BD PEF, L/s	4.7 ± 0.5^55^ ^,^ ^56^ ^,^ ^57^	7.8 ± 0.8^55^	9.3 ± 0.6^56^	8.7 ± 0.6^57^	<0.0001
Post-BD PEF, % predicted value	67.9 ± 5.6^58^ ^,^ ^59^ ^,^ ^60^	102.3 ± 5.8^58^	109.1 ± 4.3^59^	108.1 ± 4.1^60^	<0.0001
Post-BD FEV_1_/FVC, %	61.8 ± 2.1^61^ ^,^ ^62^ ^,^ ^63^	78.2 ± 2.4^61^ ^,^ ^64^	84.9 ± 2.3^62^	87.2 ± 1.0^63^ ^,^ ^64^	<0.0001
Post-BD FEF_25%–75%_, L/s	1.1 ± 0.2^65^ ^,^ ^66^ ^,^ ^67^	2.8 ± 0.3^65^ ^,^ ^68^ ^,^ ^69^	4.6 ± 0.4^66^ ^,^ ^68^	4.3 ± 0.3^67^ ^,^ ^69^	<0.0001
Post-BD FEF_25%–75%_, % predicted value	35.2 ± 3.8^70^ ^,^ ^71^ ^,^ ^72^	74.6 ± 7.1^70^ ^,^ ^73^ ^,^ ^74^	97.9 ± 6.1^71^ ^,^ ^73^	105.9 ± 5.3^72^ ^,^ ^74^	<0.0001
F_E_NO, ppb	27.0 (14.0–33.0)^75^	18.0 (11.2–38.7)	30.3 (18.6–45.5)^76^	12.3 (8.8–20.1)^75^ ^,^ ^76^	<0.01
Total serum IgE, kU/L	189.0 (75.7–393.5)^77^	102.0 (48.7–196.0)^78^ ^,^ ^79^	389.0 (204.0–687.0)^78^ ^,^ ^80^	21.9 (15.2–30.4)^77^ ^,^ ^79^ ^,^ ^80^	<0.0001
Specific serum IgE, kU/L	2.4 (0.5–39.3)^81^	11.0 (0.4–33.7)	46.2 (13.2–93.4)^81^ ^,^ ^82^	0.4 (0.3–10.2)^82^	<0.001

Abbreviations: BD, bronchodilator; FEF_F25–75%_, forced expiratory flow at 25%–75% of FVC; F_E_NO, fractional exlahed nitric oxide; FEV_1,_ forced expiratory volume in 1 s; FEV_1_/FVC%, FEV_1_ as percent of FVC; FVC, forced vital capacity; ICS, inhaled corticosteroids; IgE, immunoglobulin E; n.a., not applicable; n.s., not significant; PEF, peak expiratory flow.

a For continuous data, values are expressed as mean ± SEM, or median and interquartile range (25th and 75th percentiles), after assessing for normality with D'Agostino-Pearson omnibus normality test. Depending on data distribution, ANOVA, or Kruskal-Wallis test was used for between group comparisons and overall *p* values are shown. To reduce the chance of type I error, *post hoc* Tukey’s multiple comparisons test or *post hoc* Dunn’s multiple comparisons test was used and *p* values are reported below. Categorical data are expressed as numbers. Fisher’s exact test was used for comparing two groups; Chi square test was used for comparing more than two groups. Significance was defined as a value of *p* < 0.05.

b For continuous variables, *p* values after ANOVA or Kruskal-Wallis test for normally distributed or nonparametric data, respectively, are shown; for categorical variables, *p* values after Chi square test or Fisher’s exact test are shown.

c Atopy was assessed by measuring serum specific IgE against a panel of common aeroallergens. Subjects with asthma having serum specific IgE concentrations > 1 kU/L against the aeroallergen tested were considered atopic. ^1^ *p* < 0.01; ^2^ *p* < 0.05; ^3^ *p* < 0.001.

d Current and ex-smokers were included in the sample group and compared with non smoker group by Fisher’s exact test.

e Only severe asthma and moderate asthma groups were compared.

f Four severe asthma patients were on maintenance treatment with oral methylprednisolone (2–16 mg once daily), one severe asthma patient was on oral prednisone (12.5 mg once daily), and one severe asthma patient was on oral deflazacort (15 mg once daily).

g One missing data, severe asthma group; 4 missing data, mild asthma group; 6 missing data, mild asthma group; 4 missing data, healthy control group.

Post-hoc test *p* values.

^1^ *p* < 0.0001.

^2^ *p* < 0.001.

^3^ *p* < 0.01.

^4^ *p* < 0.05.

^5^ *p* < 0.001.

^6^ *p* < 0.05.

^7^ *p* < 0.05.

^8^ *p* < 0.05.

^9^ *p* < 0.01.

^10^ *p* < 0.0001.

^11^ *p* < 0.01.

^12^ *p* < 0.0001.

^13^ *p* < 0.0001.

^14^ *p* < 0.0001.

^15^ *p* < 0.05.

^16^ *p* < 0.0001.

^17^ *p* < 0.0001.

^18^ *p* < 0.0001.

^19^ *p* < 0.01.

^20^ *p* < 0.0001.

^21^ *p* < 0.0001.

^22^ *p* < 0.01.

^23^ *p* < 0.01.

^24^ *p* < 0.001.

^25^ *p* < 0.001.

^26^ *p* < 0.0001.

^27^ *p* < 0.0001.

^28^ *p* < 0.0001.

^29^ *p* < 0.0001.

^30^ *p* < 0.0001.

^31^ *p* < 0.001.

^32^ *p* < 0.0001.

^33^ *p* < 0.0001.

^34^ *p* < 0.001.

^35^ *p* < 0.0001.

^36^ *p* < 0.001.

^37^ *p* < 0.0001.

^38^ *p* < 0.0001.

^39^ *p* < 0.0001.

^40^ *p* < 0.001.

^41^ *p* < 0.0001.

^42^ *p* < 0.0001.

^43^ *p* < 0.0001.

^44^ *p* < 0.0001.

^45^ *p* < 0.0001.

^46^ *p* < 0.001.

^47^ *p* < 0.0001.

^48^ *p* < 0.0001.

^49^ *p* < 0.0001.

^50^ *p* < 0.0001.

^51^ *p* < 0.0001.

^52^ *p* < 0.05.

^53^ *p* < 0.0001.

^54^ *p* < 0.001.

^55^ *p* < 0.01.

^56^ *p* < 0.0001.

^57^ *p* < 0.0001.

^58^ *p* < 0.0001.

^59^ *p* < 0.0001.

^60^ *p* < 0.0001.

^61^ *p* < 0.0001.

^62^ *p* < 0.0001.

^63^ *p* < 0.0001.

^64^ *p* < 0.05.

^65^ *p* < 0.001.

^66^ *p* < 0.0001.

^67^ *p* < 0.0001.

^68^ *p* < 0.01.

^69^ *p* < 0.01.

^70^ *p* < 0.0001.

^71^ *p* < 0.0001.

^72^ *p* < 0.0001.

^73^ *p* < 0.05.

^74^ *p* < 0.01.

^75^ *p* < 0.05.

^76^ *p* < 0.05.

^77^ *p* < 0.0001.

^78^ *p* < 0.05.

^79^ *p* < 0.05.

^80^ *p* < 0.0001.

^81^ *p* < 0.05.

^82^ *p* < 0.0001.

Participants were matched for gender and smoking habit, but not for age (*p* < 0.0001) and prevalence of atopy (*p* = 0.008) (Table 1). Age was elevated in severe asthma adults compared with mild asthma adults (*p* < 0.0001) and healthy control subjects (*p* < 0.001), but not compared with moderate asthma adults (Table 1). All adults with mild asthma were atopic (Table 1). The prevalence of atopy was similar across the other study groups (*p* = 0.65).

Supplementary Table S1 shows important features identified by one-way ANOVA and Fisher’s LSD *post hoc* test.

There was no difference in serum FLC concentrations in women and men in each study groups (Supplementary Table S2).

### Ig FLC concentrations in serum

**FIGURE 2:**
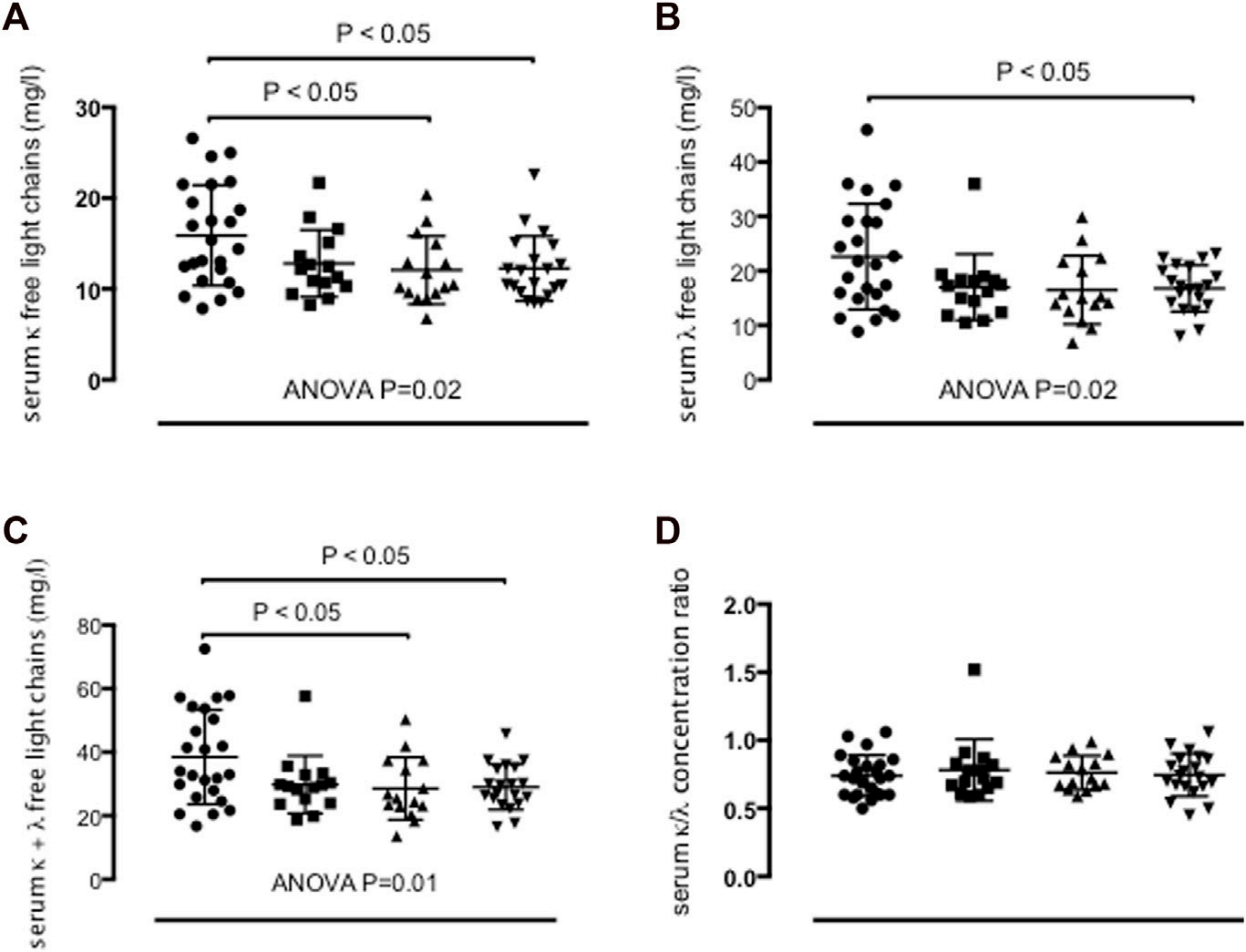
Concentrations of κ free light chains (FLC) **(A)**, λ FLC **(B)**, sum of κ and λ FLC **(C)**, and κ/λ concentration ratio **(D)** in serum in persistent severe asthma (*n* = 24) (dots), persistent moderate asthma (*n* = 15) (squares), persistent mild asthma (*n* = 15) (up-pointing triangles) and healthy control subjects (*n* = 20) (down-pointing triangles). Mean values with SD are shown. *p* < 0.05 was considered significant.

Severe asthma adults (15.9 ± 5.5 mg/L, ANOVA *p*-value = 0.02), but not moderate asthma adults (12.8 ± 3.7 mg/L) showed higher serum κ FLC concentrations than mild asthma adults (12.1 ± 3.7 mg/L, *p* < 0.05) and healthy control adults (12.3 ± 3.6 mg/L, *p* < 0.05) (Table 2; Figure 2A). Serum λ FLC concentrations were elevated in severe asthma individuals (22.6 ± 9.7 mg/L, ANOVA *p*-value = 0.02) compared with healthy control individuals (16.8 ± 4.3 mg/L, *p* < 0.05) (Table 2; Figure 2B). Compared with mild asthma adults (28.6 ± 9.8 mg/L, *p* < 0.05) and healthy control individuals (29.1 ± 7.2 mg/L, *p* < 0.05), the sum of κ and λ FLC concentrations in serum was elevated in severe asthma adults (38.6 ± 14.9 mg/L, ANOVA *p*-value 0.01), but not in moderate asthma adults (29.8 ± 9.1 mg/L) (Table 2; Figure 2C). κ/λ serum FLC concentration ratio was similar across study groups (Table 2; Figure 2D). Using one-way ANOVA and Fisher’s LSD *post hoc* test, instead of Tukey’s *post hoc* test, both types of FLC in serum were significantly elevated in severe asthma adults compared with all the other study groups (κ: f value = 3.6169, *p*-value = 0.017266, adjusted *p* values (FDR) = 0.020463; λ: f value = 3.6184, *p*-value = 0.017235; FDR = 0.020463) (Supplementary Table S1, anova_posthoc.xlsx supplementary file).

**TABLE 2:** Serum Free Light Chain (FLC) concentrations in study groups^a^.

	Severe asthmatics (*n* = 24)	Steroid-treated moderate asthmatics (*n* = 15)	Steroid-naïve mild asthmatics (*n* = 15)	Healthy control subjects (*n* = 20)	ANOVA *p* value
κ FLC, mg/L	15.9 ± 5.5^b^ ^,^ ^c^	12.8 ± 3.7	12.1 ± 3.7^b^	12.3 ± 3.6^c^	0.02
λ FLC, mg/L	22.6 ± 9.7^d^	17.0 ± 6.1	16.5 ± 6.3	16.8 ± 4.3^d^	0.02
κ/λ ratio, %	0.7 ± 0.2	0.8 ± 0.2	0.8 ± 0.1	0.7 ± 0.2	0.86
κ + λ, mg/L	38.6 ± 14.9^e^ ^,^ ^f^	29.8 ± 9.1	28.6 ± 9.8^e^	29.1 ± 7.2^f^	0.01

Abbreviations: FLC, free light chain.

a Data are expressed as mean ± SD., Data were normally distributed after log transformation. Frequency of distribution was assessed with the D'Agostino-Pearson omnibus normality test. One-way ANOVA, with *post hoc* Tukey’s multiple comparisons test was used for between group comparison. Significance was defined as a value of *p* < 0.05.

*Post-hoc* *p* values.

b *p* < 0.05.

c *p* < 0.05.

d *p* < 0.05.

e *p* < 0.05.

f *p* < 0.05.

Supplementary Figure S1 shows ROC curves for serum κ and λ FLC measurements in severe asthma *versus* healthy control individuals and severe asthma *versus* mild asthma individuals. Measurement of serum κ FLC concentrations distinguished severe asthma individuals from either healthy individuals (Supplementary Datasheet S1) or mild asthma individuals (Supplementary Datasheet S2) with an area under the ROC curve (AUROC) of 0.71 for both, with *p* values of 0.02 and 0.03, respectively (Supplementary Figures S1A, B). Measurement of serum λ FLC concentrations distinguished severe asthma individuals from either healthy individuals (Supplementary Datasheet S3) or mild asthma individuals (Supplementary Datasheet S4) with an area under the ROC curve (AUROC) of 0.66 (*p* = 0.066) and 0.69 (*p* = 0.046), respectively (Supplementary Figures S1C, D).

**FIGURE 3:**
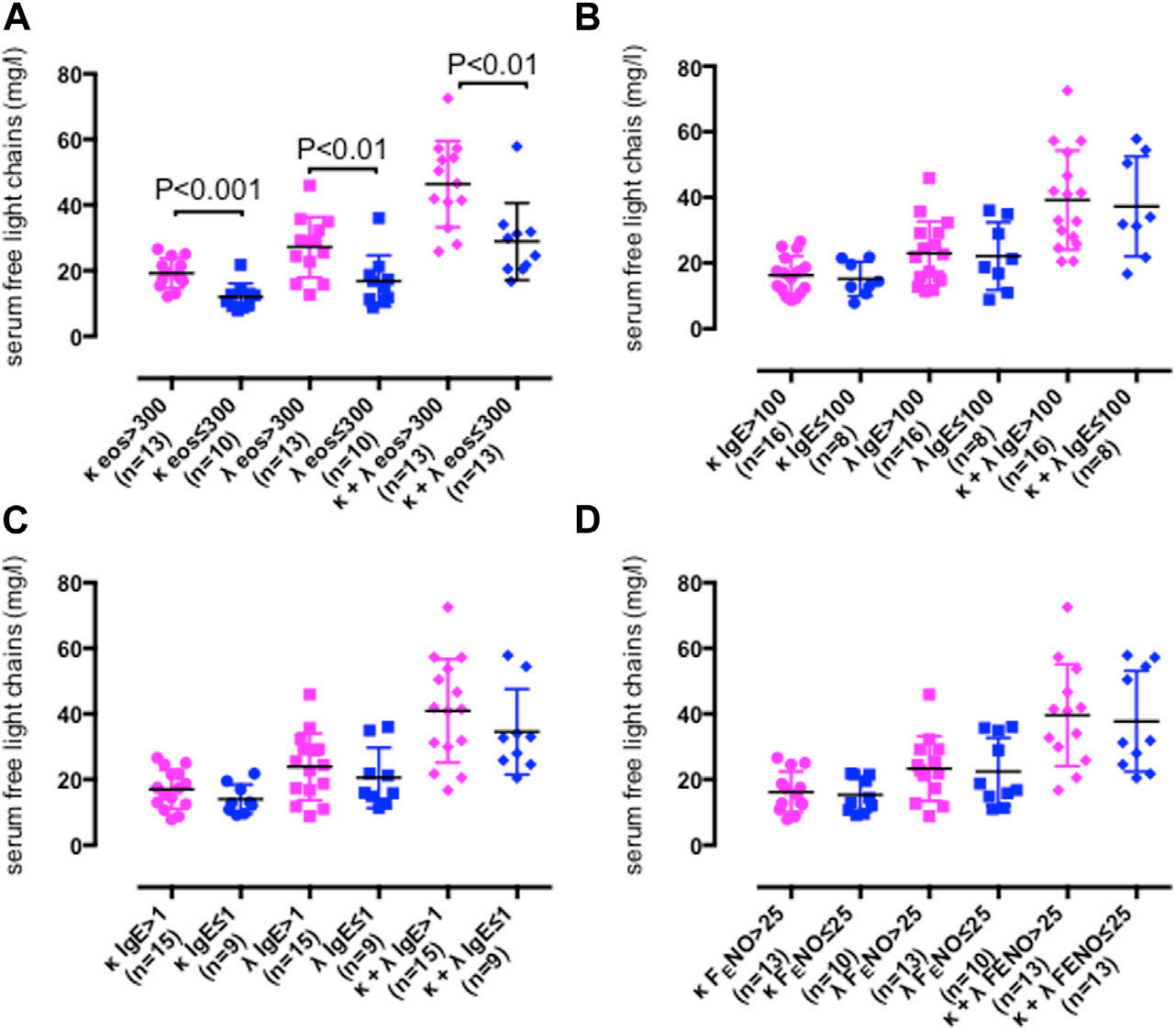
Serum free light chain (FLC) concentrations in persistent severe asthma participants with peripheral blood eosinophil counts > 300/μL (κ, pink dots; λ, pink squares, κ and λ concentration sum, pink diamonds) or ≤ 300/μL (κ, blue dots; λ, blue squares, κ and λ concentration sum, blue diamonds) **(A)**, serum total IgE concentrations > 100 kU/L (κ, pink dots; λ, pink squares, κ and λ concentration sum, pink diamonds) or ≤ 100 kU/L (κ, blue dots; λ, blue squares, κ and λ concentration sum, blue diamonds) **(B)**, serum specific IgE > 1 (κ, pink dots; λ, pink squares, κ and λ concentration sum, pink diamonds) or ≤ 1 (κ, blue dots; λ, blue squares, κ and λ concentration sum, blue diamonds) **(C)** or fractional exhaled nitric oxide (F_E_NO) > 25 ppb (κ, pink dots; λ, pink squares, κ and λ concentration sum, pink diamonds) or ≤ 25 ppb (κ, blue dots; λ, blue squares, κ and λ concentration sum, blue diamonds) **(D)**. Mean values with SD are shown. *p* < 0.05 was considered significant.

Severe asthma adults with peripheral blood eosinophil cell counts > 300 cells/µL showed higher serum κ (*p* < 0.001) and λ FLC concentrations (*p* < 0.01) than severe asthma adults with peripheral blood eosinophil cell counts ≤ 300 cells/µL (Table 3; Figure 3A), whereas serum κ and λ FLC concentrations were similar across study groups with high or low serum total IgE (Table 3; Figure 3B), with or without atopy as measured by serum specific IgE (Table 3; Figure 3C), or high or low F_E_NO concentrations (Table 3; Figure 3D).

**TABLE 3:** Serum Free Light Chain (FLC) concentrations in severe asthma subgroups based on T2 high or T2 low surrogate markers^a^.

	Blood eosinophils (cells/µL)		*p*-value	Total serum IgE (kU/mL)		*p*-value	Specific serum IgE (kU/mL)		*p*-value	F_E_NO (ppb)		*p*-value
	>300	≤300		>100	≤100		>1	≤1		>25	≤25	
n	13	10		16	8		15	9		13	10	
k FLC, mg/L	19.2 ± 4.5	12.1 ± 4.0	0.001	16.3 ± 5.8	15.2 ± 5.2	0.67	17.0 ± 5.9	14.0 ± 4.4	0.20	16.2 ± 6.1	15.3 ± 5.2	0.71
λ FLC, mg/L	27.2 ± 9.2	16.8 ± 7.8	0.01	22.9 ± 9.7	22.1 ± 10.3	0.85	23.9 ± 10.1	20.5 ± 9.1	0.42	23.3 ± 9.8	22.4 ± 10.3	0.84
k/λ ratio, %	0.7 ± 0.2	0.8 ± 0.1	0.87	0.7 ± 0.2	0.74 ± 0.2	0.97	0.7 ± 0.1	0.7 ± 0.2	0.86	0.7 ± 0.1	0.7 ± 0.1	0.98
k + λ, mg/L	46.4 ± 13.1	28.9 ± 11.8	0.005	39.2 ± 15.1	37.3 ± 15.2	0.78	40.9 ± 15.8	34.6 ± 13.0	0.32	39.5 ± 15.6	37.8 ± 15.4	0.79

Abbreviations: F_E_NO, fractional exhaled nitric oxide; FLC, free light chain; Ig, immunoglobulin.

The eosinophilic phenotype was defined based on peripheral blood eosinophil cell counts ≥ 300 cells/µL.

Atopy was assessed by measuring serum specific IgE against a panel of common aeroallergens. Subjects with severe asthma having serum specific IgE concentrations > 1 kU/L against the aeroallergen tested were considered atopic.

Elevated or non elevated serum total IgE was defined based on serum total IgE concentrations > 100 kU/L or ≤ 100 kU/L.

a Data are expressed as mean ± SD, Data were normally distributed after log transformation. Frequency of distribution was assessed with the D'Agostino-Pearson omnibus normality test. Unpaired *t*-test was used for between group comparison. Significance was defined as a value of *p* < 0.05.

### T2 inflammatory outcomes

#### Peripheral blood eosinophils

**FIGURE 4:**
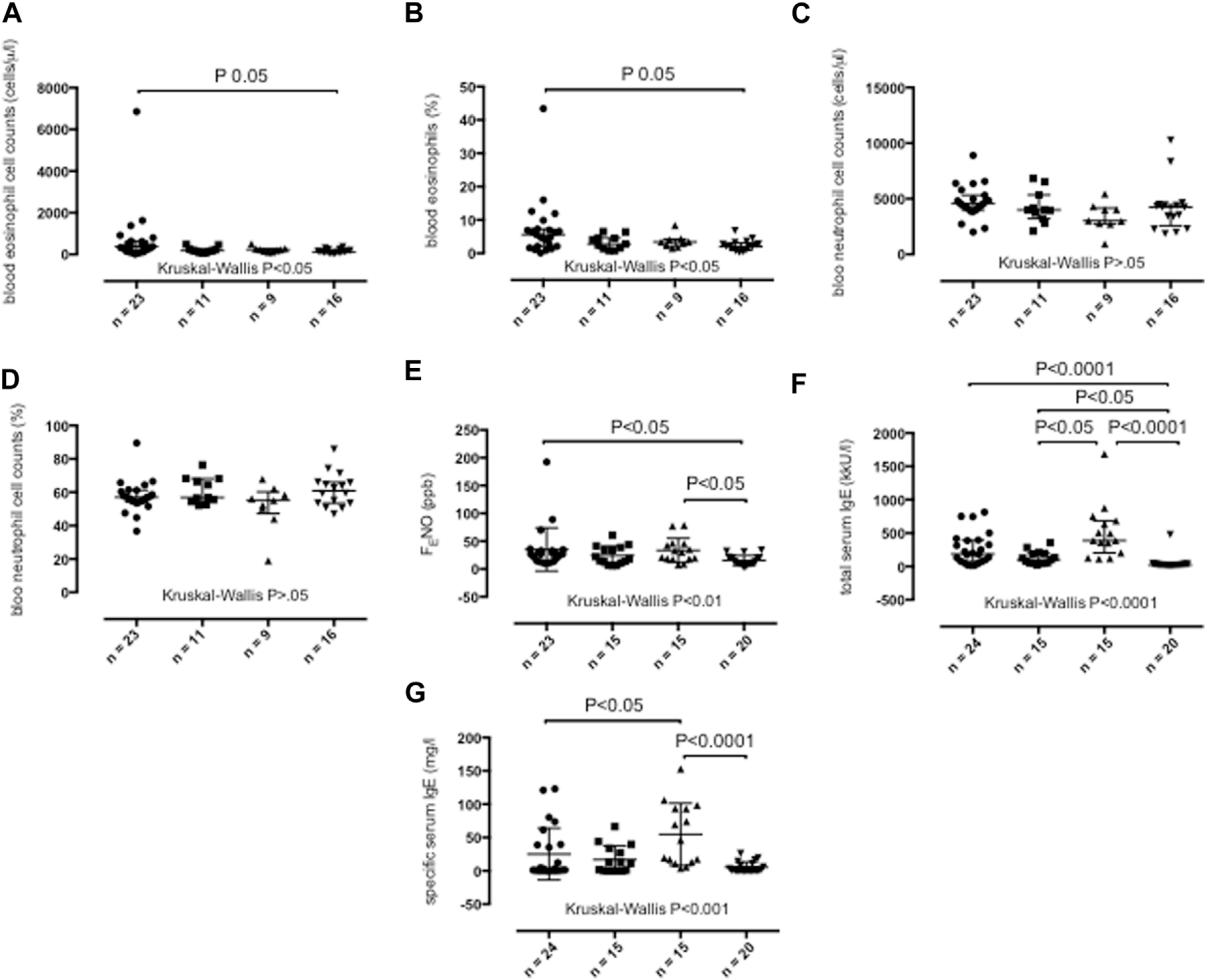
Peripheral blood absolute eosinophil cell counts **(A)**, peripheral blood percentage eosinophil cell counts **(B)**, peripheral blood absolute neutrophil cell counts **(C)**, peripheral blood percentage neutrophil cell counts **(D)**, fractional exhaled nitric oxide (F_E_NO) concentrations **(E)**, total serum IgE concentrations **(F)** and specific serum IgE concentrations **(G)** in persistent severe asthma (dots), persistent moderate asthma (squares), persistent mild asthma (up-pointing triangles) and healthy control subjects (down-pointing triangles). Median values with interquartile range are shown. *p* < 0.05 was considered significant.

Compared with healthy control subjects, peripheral blood eosinophil cell counts as absolute numbers (*p* < 0.05) (Figure 4A) and as a percentage of total blood cell counts (*p* < 0.05) (Figure 4B) were elevated in severe asthma adults (Table 1). Peripheral blood neutrophil cell counts across study groups were similar (Table 1; Figures 4C, D).

#### F_E_NO

Severe asthma adults (*p* < 0.05) and mild asthma adults (*p* < 0.05) (Figure 4E) showed higher F_E_NO values than healthy participants (Table 1).

#### Serum IgE

Serum total IgE were elevated in individuals with severe asthma (*p* < 0.0001), moderate asthma (*p* < 0.05) and mild asthma (*p* < 0.0001) compared with healthy participants and in individuals with mild asthma compared with those with moderate asthma (*p* < 0.05) (Table 1; Figure 4F). Mild asthma adults showed higher serum specific IgE concentrations than severe asthma (*p* < 0.05) and healthy adults (*p* < 0.0001) (Table 1; Figure 4G).

### Pulmonary function testing

Adults with severe asthma had lower pre-bronchodilator (Supplementary Figure S2) and post-bronchodilator lung function test values (Supplementary Figure S3) than moderate asthma, mild asthma and healthy control adults (Table 1). Compared with mild asthma and healthy control adults (Table 1), moderate asthma adults showed lower pre-bronchodilator (Supplementary Figure S2) and post-bronchodilator lung function test values (Supplementary Figure S3), Persistent mild asthma and healthy control adults showed similar lung function test values (Table 1, Supplementary Figures S2, S3).

### Correlations

**FIGURE 5:**
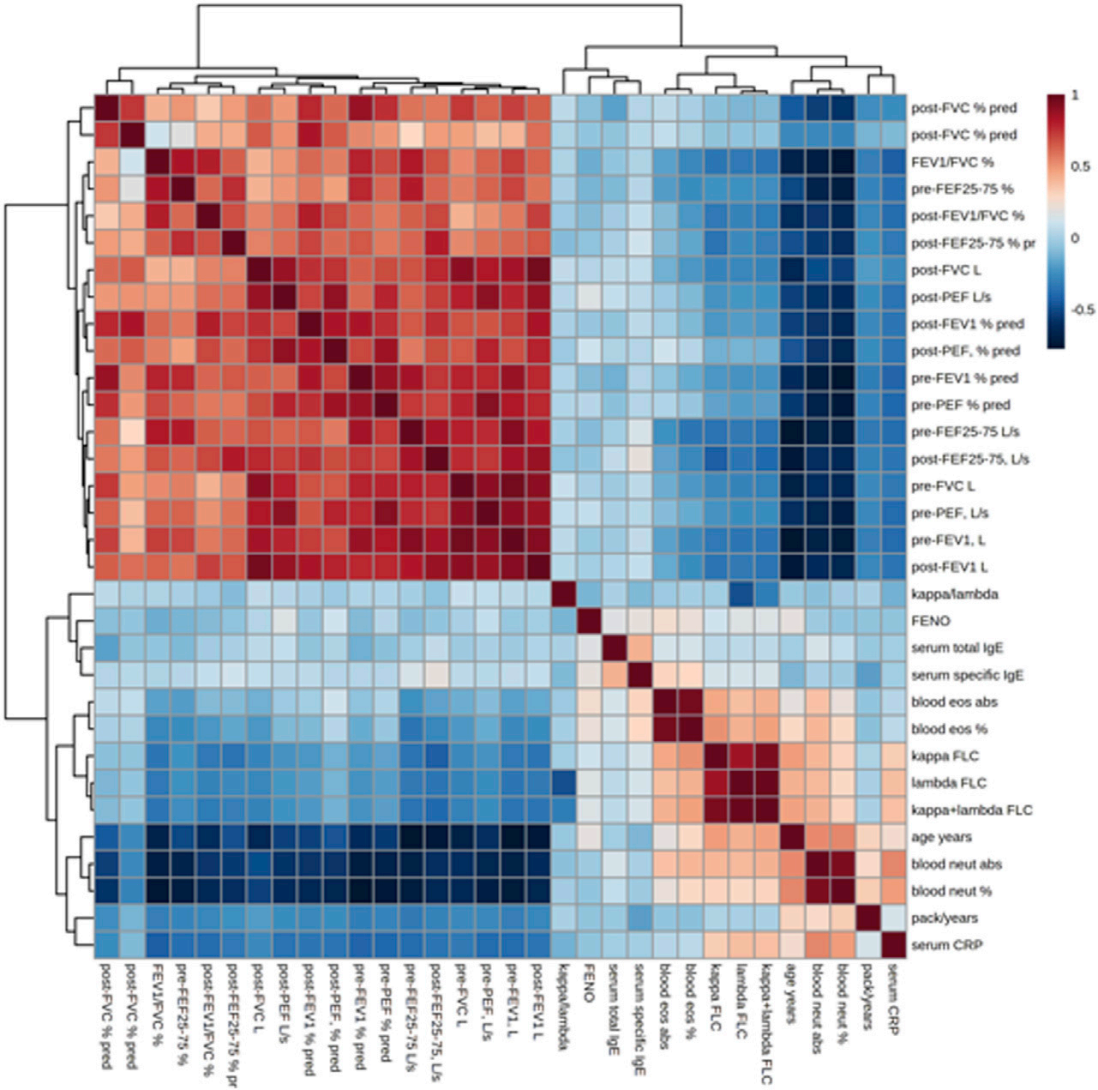
Heatmap showing the correlations between study variables, including serum κ and λ free light chain (FLC) concentrations, pulmonary function tests, and inflammatory outcomes. Positive correlations are shown in red; negative correlations are shown in blue.

**FIGURE 6:**
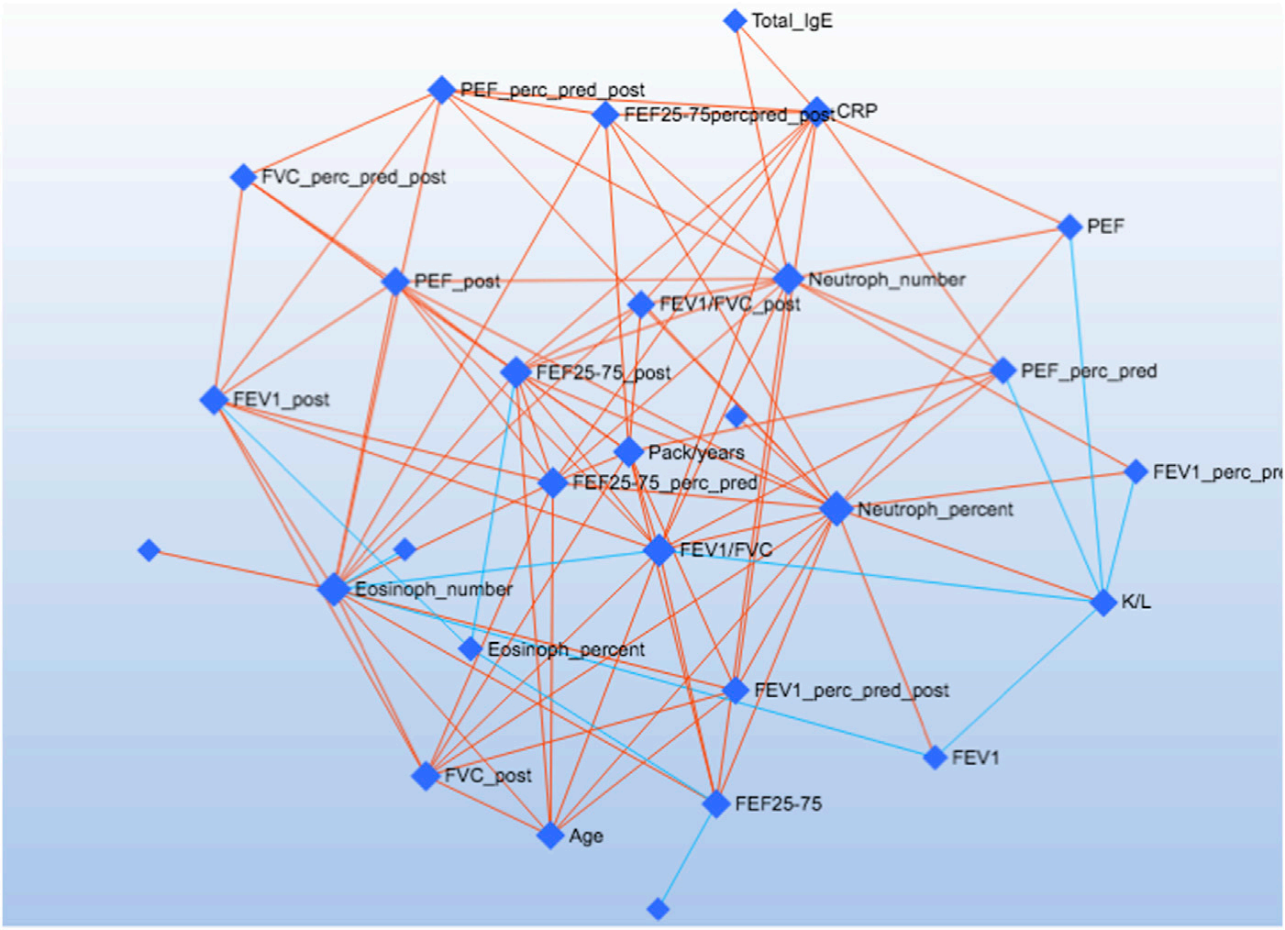
The Debiased Sparse Partial Correlation (DSPC) network. The nodes are input variables, while the edges represent the association measures. For better visualization, the default DSPC network only shows the top correlations (edges) based on their *p*-value rankings (top 20% when the total number of edges is less than 1000).

**FIGURE 7:**
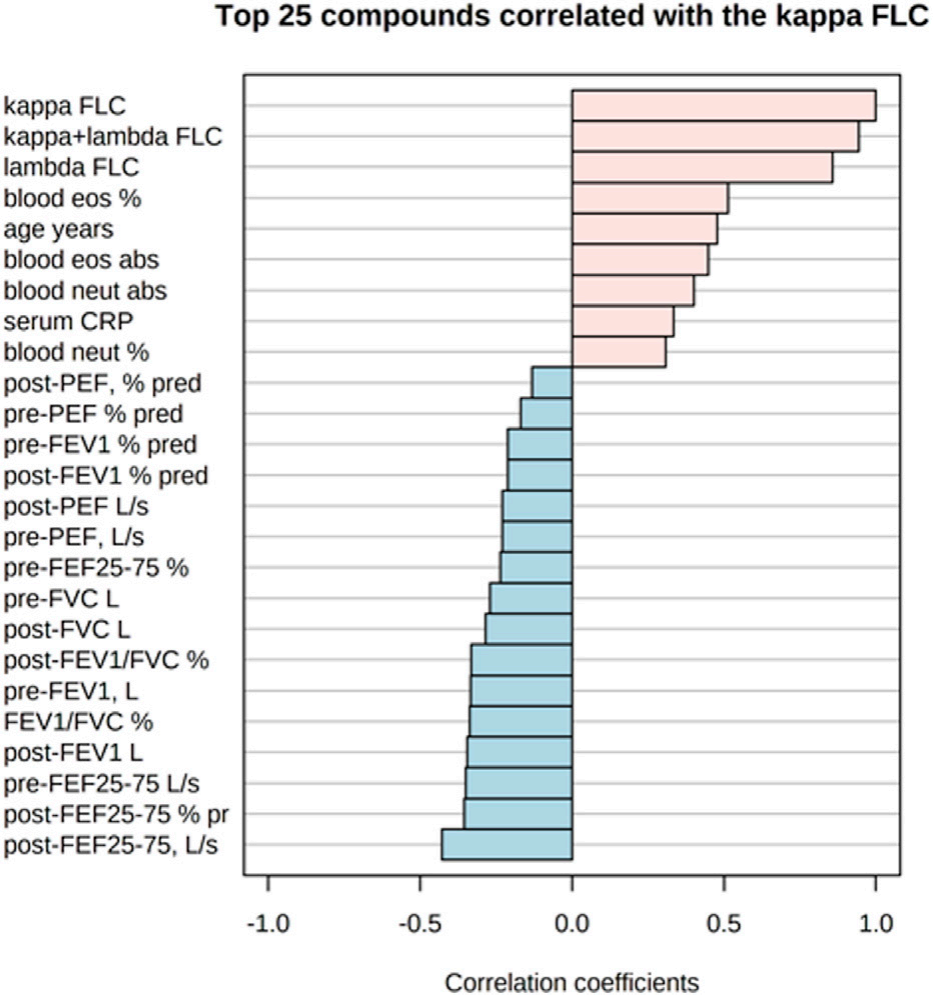
Top 25 study variables correlated with serum κ free light chain (FLC) concentrations. Values of Pearson’s correlation coefficients are shown.

**FIGURE 8:**
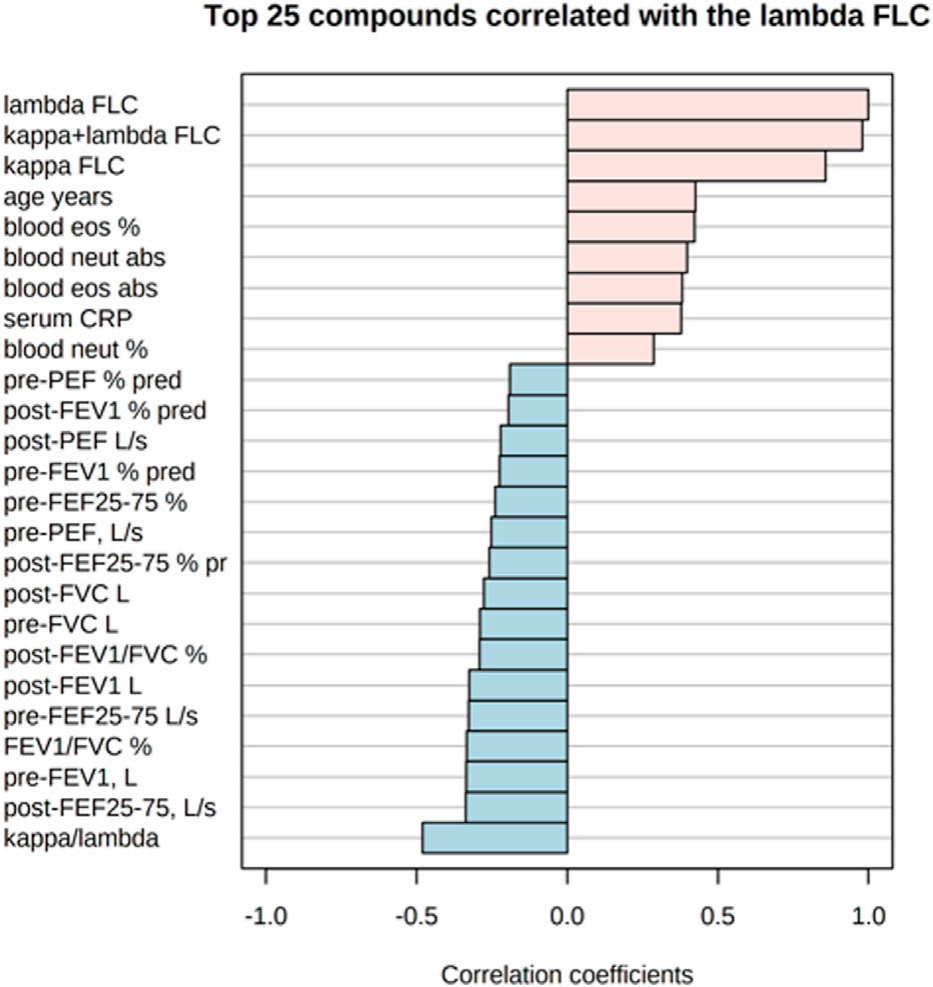
Top 25 study variables correlated with serum λ free light chain (FLC) concentrations. Values of Pearson’s correlation coefficients are shown.

Figure 5 shows the correlations of the study outcomes as a heatmap. Figure 6 shows the correlation network. Serum κ and λ FLC concentrations were highly correlated and correlated with age (Figure 5; Figure 7; Figure 8, Supplementary Figure S4; Supplementary Tables S3, S4; correlation matrix and anova_posthoc Excel files, online supplement). A negative correlation was observed between serum κ and λ FLC concentrations and most pulmonary function testing outcomes (Figure 5; Figure 7; Figure 8, Supplementary Figure S4; Supplementary Tables S3, S4; correlation table and *p* values correlation table supplementary files).

In adults with persistent severe asthma, serum κ and λ FLC were highly correlated with inflammatory outcomes reflecting T2 high endotype, including peripheral blood eosinophils as percentage of total counts and absolute values, with peripheral blood neutrophils as percentage of total counts and absolute values, and with serum CRP concentrations (Figure 7; Figure 8, Supplementary Figure S4, Supplementary Tables S3, S4; correlation table and *p* values correlation table supplementary files). By contrast, serum FLC concentrations were not correlated with other surrogate markers reflecting T2 high airway inflammation, including F_E_NO, serum total IgE or specific IgE (Supplementary Tables S3, S4; correlation table and *p* values correlation table supplementary files). Correlations of κ FLC or λ FLC serum concentrations with peripheral blood lymphocytes and monocytes are shown in Supplementary Figures S5, S6, respectively. Linear regression analyses of κ FLC or λ FLC serum concentrations and peripheral blood lymphocytes and monocytes are shown in Supplementary Figures S7, S8, respectively. Serum FLC concentrations were correlated with peripheral blood lymphocyte absolute values (κ FLC: Pearson *r* = 0.45, *p* < 0.05, *n* = 23 (Supplementary Figure S5A); λ FLC: Pearson *r* = 0.53, *p* < 0.05, *n* = 23 (Figure 6A), but not with lymphocytes as percentage of total cell counts (Supplementary Figures S5B, S6B), monocyte absolute values (Supplementary Figures S5C, S6C) or monocytes as percentage of total cell count (Supplementary Figures S5D, S6D).

## Discussion

In this study, we show that κ and λ FLC concentrations in serum are elevated in adults with severe persistent asthma, but not in those with moderate or mild persistent asthma, compared with healthy control adults. Elevated serum FLC concentrations in severe asthma adults who were on maintenance therapy with high dose of ICS and/or oral corticosteroids suggest that serum FLC concentrations are relatively resistant to these drugs. However, prospective randomised controlled powered studies are required to clarify the effects of pharmacotherapies, including glucocorticoid and biologic therapies, on serum FLC in persons with severe persistent asthma. Our study groups were not matched for allergy, as all mild asthma adults were atopic, and for age, mild asthma and healthy individuals being younger than those with severe asthma. However, serum FLC concentrations seem to be independent of atopy, as their values were normal in mild asthma adults, were similar in atopic or nonatopic severe asthma individuals, were similar in severe asthma individuals irrespective of serum total IgE (≥100 kU/L), and were not correlated with total serum IgE or serum specific IgE. The potential age-dependency of serum FLC concentrations has been formally addressed in the original article which first reported on serum reference intervals and diagnostic ranges for κ and λ FLC (Katzmann et al., 2002). Age dependence in 282 reference serum samples from individuals aged from 21 to 90 years was not obesrved for both κ and λ FLC (Katzmann et al., 2002). Although serum FLC concentrations tended to increase with age, this trend was not significant (Katzmann et al., 2002). For this reason, the defined central 95% reference interval was not adjusted for age (Katzmann et al., 2002). Another study reported elevated serum concentrations of κ FLC in both atopic (mean age 35.4 years) and nonatopic asthmatics (mean age 30.7 years) compared with nonasthmatic individuals (mean age 36.6 years) (Kraneveld et al., 2005). Taken together, this evidence suggests that age does not seem to have a major role in determining elevation of serum FLC in individuals with severe asthma. However, the potential effect of age on serum FLC concentrations in individuals with severe asthma should be specifically addressed in future studies.

In adults with persistent asthma, we observed correlation between both types of FLC in serum and inflammatory outcomes, such as peripheral blood eosinophils, which reflect T2 high inflammation, peripheral blood neutrophils and serum CRP concentrations. Moreover, we did not observe any correlation between serum FLC concentrations and other surrogate markers of T2 high inflammation, including FENO, serum total IgE or serum specific IgE. Taken together, these findings support previous work showing increased serum κ FLC concentration in adults with asthma compared with healthy adults irrespective of atopy (Kraneveld et al., 2005). These data suggest that serum FLC concentrations are correlated with T2 low inflammatory outcome measures. Regarding T2 high inflammatory outcomes, peripheral blood eosinophil cell counts were elevated in adults with severe asthma compared with healthy adults, but not in comparison with mild or moderate asthma adults. The correlations of serum κ and λ FLC concentrations with peripheral blood eosinophils, but not with other T2 high inflammatory outcomes, including F_E_NO and serum total and specific IgE, might reflect, at least partially, the heterogeneity of the peripheral blood eosinophilic population. Using sputum proteomic profiling in adults with severe asthma, three sub-populations of sputum eosinophils have been identified in a previous study (Schofield et al., 2019). In addition to that, many anti-IL5 therapies have not achieved a complete therapeutic response in many persons with severe eosinophilic asthma despite fulfilling the requisite biomarker criteria (Haldar et al., 2011; Pavord et al., 2012; Bel et al., 2014; Ortega et al., 2014; Chupp et al., 2017), that is, peripheral blood eosinophil counts equal to or higher that 150 cells/µL. The correlation between peripheral blood eosinophil absolute cell counts and peripheral blood neutrophil cell counts, which do not reflect T2 high inflammation, observed in our study, is consistent with the postulated heterogeneity of the peripheral blood eosinophilic population. However, further research to formally address the potential existence of eosinophilic sub-population(s) associated with T2 low inflammation is required.

Serum κ and λ FLC concentrations were negatively correlated with most spirometric parameters, including FEF_25%–75%_, which reflect peripheral airway function. Lung CT scan studies aiming at investigating the relationships between serum Ig FLC concentrations and peripheral airways structural changes in persons with severe asthma are required.

Previous studies have shown that Ig FLC mediate antigen-specific mast cell hypersensitivity responses through activation of a FLC receptor on mast cells, suggesting that these molecules might be involved in the pathophysiology of adult asthma (Redegeld et al., 2002; Kraneveld et al., 2005; Mortaz et al., 2017). Some authors postulate that the production of FLC might provide a functional link between the mast cell and the development of non-allergic responses and might be a novel factor in the humoral immune response to antigen exposure (Redegeld et al., 2002; Kraneveld et al., 2005; Mortaz et al., 2017).

Both κ and λ FLC share a common binding site on Tamm–Horsfall protein (THP), a monomeric glycoprotein produced by cells in the ascending limb of Henle of the kidney (Huang and Sanders, 1997). The highly selective FLC antagonist (F-991), which prevents FLC binding to THP, attenuates hypersensitivity responses in various animal models and has the potential for being used as a new treatment for human allergic disease (Redegeld et al., 2002; Mortaz et al., 2017). Moreover, the presence of FLC in both atopic and nonatopic diseases might provide an alternative approach to the treatment of these diseases (Kraneveld et al., 2005).

Our study was unable to assess a potential role of FLC in the pathophysiology of asthma, which is currently unknown. The increase in serum FLC concentrations of approximately 25% observed in severe asthma individuals compared with healthy subjects is unlikely to have profound biological effects, such as increasing mast cell activation, and to be clinically significant as mast cell stimuli usually require log10 changes in stimulus to show dose-responsiveness. Mechanistic studies and/or studies aiming at measuring FLC concentrations in biological fluids more likely to reflect concentrations of these biomolecules in the respiratory system, e.g., bronchoalveolar lavage or sputum supernatants, could clarify a potential functional involvement of FLC in severe asthma.

Compared with previously published articles, the original aspects of our study include measurement of serum κ and λ Ig FLC concentrations in both men and women with different degree of asthma severity; the inclusion of a group of asthma adults who were not being treated with ICS; the assessment of the relationships between serum Ig FLC concentrations and inflammatory outcomes, including F_E_NO, total and specific serum IgE, and peripheral blood eosinophils, which are surrogate markers of T2 high pathways of asthma, serum CRP and peripheral blood neutrophils in adults with severe asthma; a machine learning-based approach to data analysis.

Study limitations are represented by the relatively small size of study groups, which requires larger powered studies to confirm these findings, and the lack of age and atopy matching across study groups. However, the size of our study, including 74 individuals, is similar to that of a previous study aiming at measuring serum FLC in asthma, which included 80 individuals, 31 individuals with asthma (17 atopics, 14 non-atopics) and 49 healthy individuals (15 atopics, 34 non-atopics) (Kraneveld et al., 2005). Future work in this research area includes measurement of FLC concentrations in urine, which is non-invasive, or sputum supernatants, which is semi-invasive, in individuals with different asthma severity.

In conclusions, serum polyclonal Ig FLC are elevated in persons with persistent severe asthma and might represent a new surrogate inflammatory marker. However, larger studies are required to formally address this point. Further research is required to clarify a potential functional involvement of FLC production in the pathophysiology of severe asthma, their association with severe asthma sub-phenotypes and endotypes and their potential utility for the assessment of the effects of pharmacotherapies for severe asthma.

## Data Availability

The original contributions presented in the study are included in the article/Supplementary Material, further inquiries can be directed to the corresponding author.
