# Supplementary figures and images for: Elevated serum polyclonal immunoglobulin free light chains in patients with severe asthma
Source: Front Pharmacol. 2023 Jun 16;14:1126535. doi: 10.3389/fphar.2023.1126535 (PMC10311563; doi:10.3389/fphar.2023.1126535)

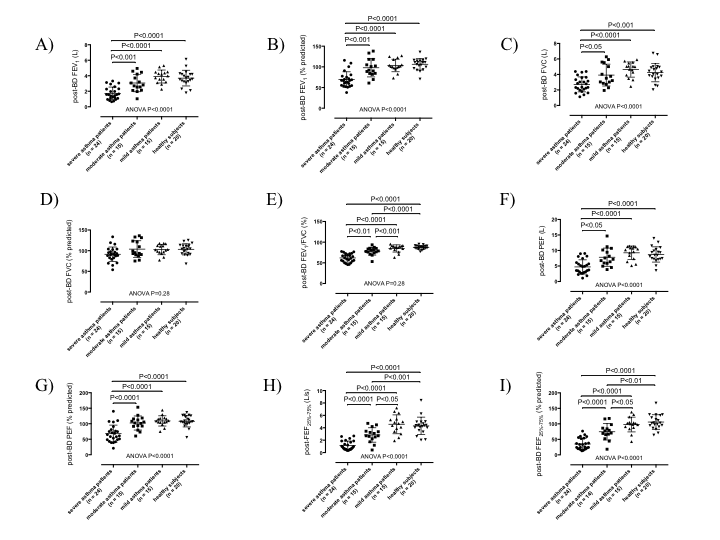

Supplement: Supplementary file 1 [file Image3.tiff]

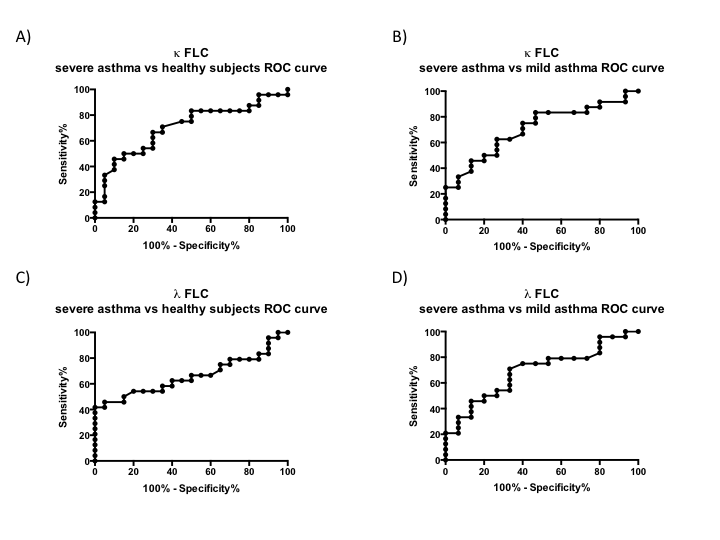

Supplement: Supplementary file 2 [file Image1.tiff]

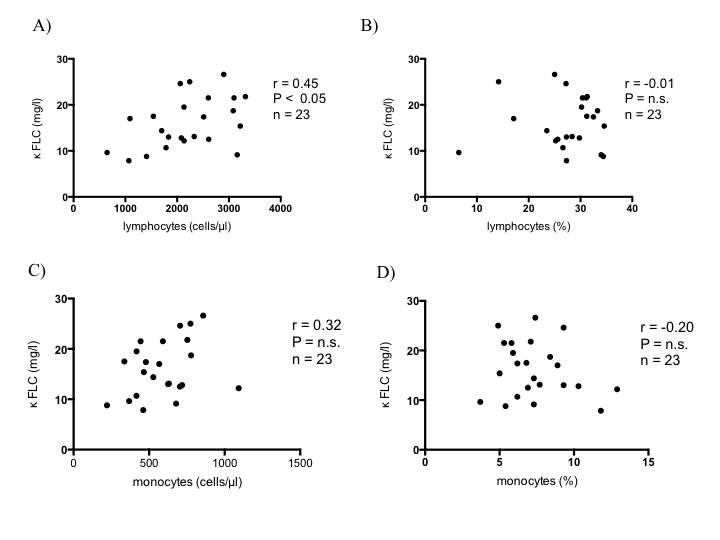

Supplement: Supplementary file 7 [file Image5.tiff]

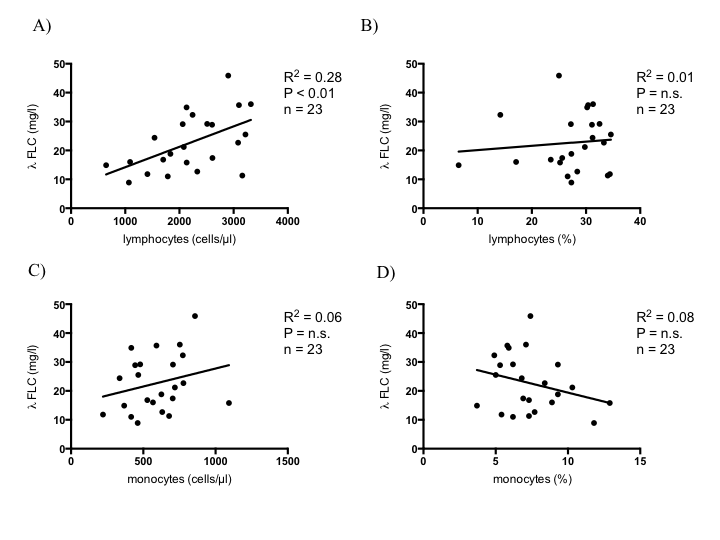

Supplement: Supplementary file 8 [file Image8.tiff]

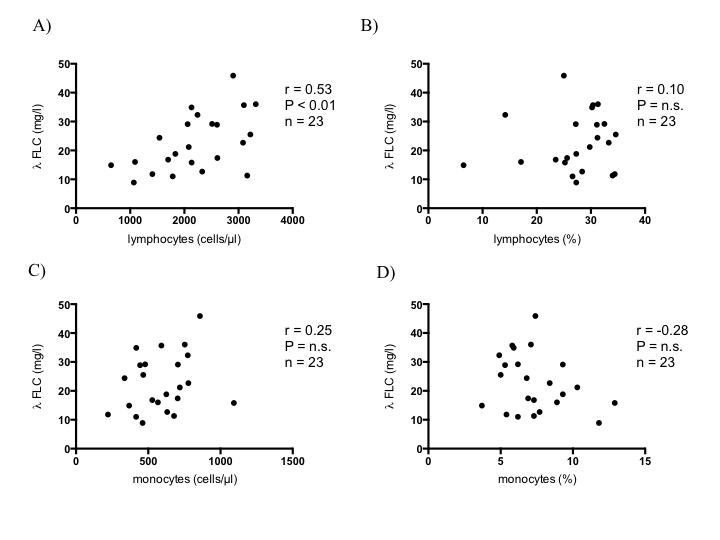

Supplement: Supplementary file 13 [file Image6.tiff]

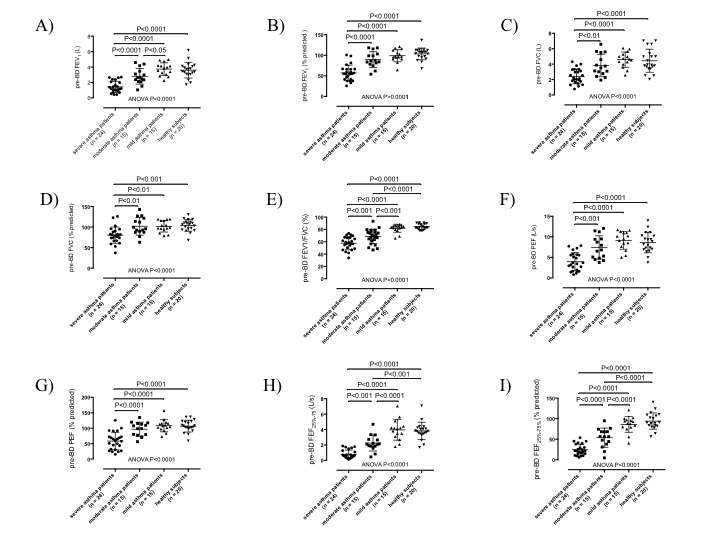

Supplement: Supplementary file 15 [file Image2.tiff]

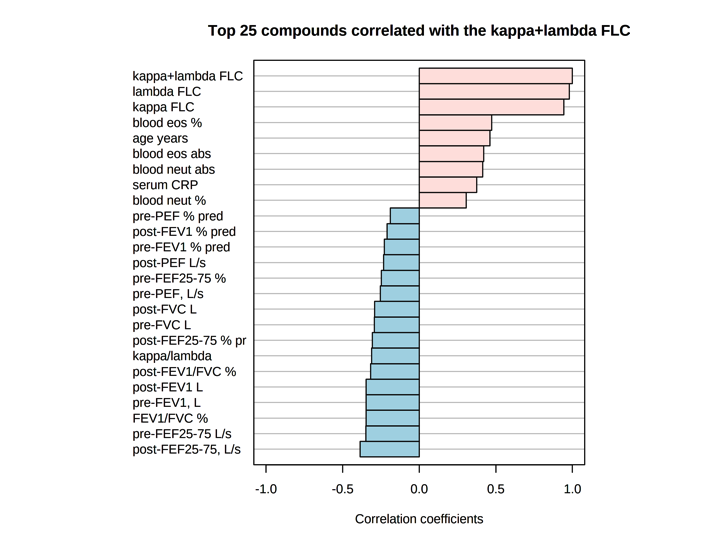

Supplement: Supplementary file 16 [file Image4.tiff]

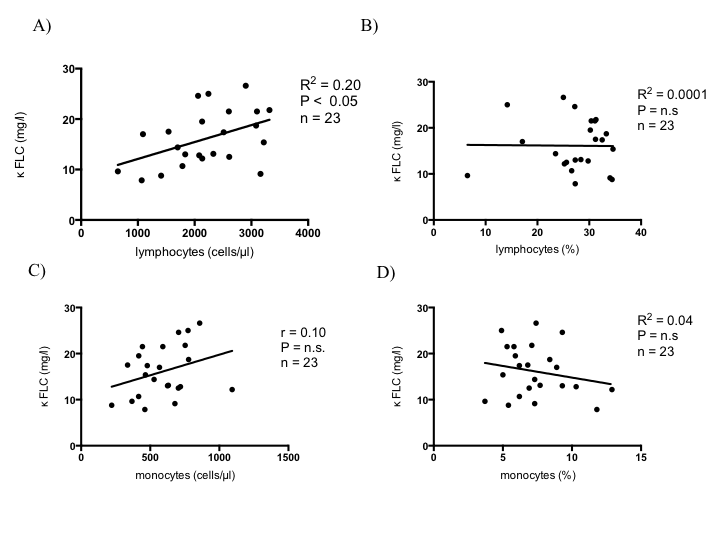

Supplement: Supplementary file 18 [file Image7.tiff]
