# Supplementary material for: Elevated serum polyclonal immunoglobulin free light chains in patients with severe asthma
Source: Front Pharmacol. 2023 Jun 16;14:1126535. doi: 10.3389/fphar.2023.1126535 (PMC10311563; doi:10.3389/fphar.2023.1126535)
Supplement: Supplementary file 4 [file Table1.docx]

**Table S1.** Important features identified by one-way ANOVA and post-hoc analysis

| Outcome measure | F value | P value | -log10(P) | FDR | Post-hoc tests |
| --- | --- | --- | --- | --- | --- |
| Pre-BD FEV1/FVC, % | 45.549 | 1.9397E-16 | 15.712 | 1.5703E-15 | 2 - 1; 3 - 1; 4 - 1; 3 - 2; 4 – 2 |
| Pre-BD FEF_25%-75%_, L | 45.526 | 1.9629E-16 | 15.707 | 1.5703E-15 | 2 - 1; 3 - 1; 4 - 1; 3 - 2; 4 – 2 |
| Pre-BD FEF_25%-75%_, % predicted value | 45.062 | 2.4827E-16 | 15.605 | 1.5889E-15 | 2 - 1; 3 - 1; 4 - 1; 3 - 2; 4 - 2; 4 – 3 |
| Pre-BD FEV_1_, % predicted value | 33.301 | 1.7349E-13 | 12.761 | 9.2528E-13 | 2 - 1; 3 - 1; 4 - 1; 4 – 2 |
| Pre-BD FEV_1_, L | 29.905 | 1.4785E-12 | 11.83 | 6.7589E-12 | 2 - 1; 3 - 1; 4 - 1; 3 - 2; 4 – 2 |
| Pre-BD PEF, % predicted value | 22.504 | 2.6142E-10 | 9.5827 | 1.0457E-9 | 2 - 1; 3 - 1; 4 – 1 |
| Post-BD FEF_25%-75%,_ L | 21.045 | 7.9785E-10 | 9.0981 | 2.8368E-9 | 2 - 1; 3 - 1; 4 - 1; 3 - 2; 3 – 4 |
| Pre-BD PEF, L | 20.323 | 1.4042E-9 | 8.8526 | 4.4934E-9 | 2 - 1; 3 - 1; 4 – 1 |
| Post-BD FEV_1_, L | 17.5 | 1.4018E-8 | 7.8533 | 4.078E-8 | 2 - 1; 3 - 1; 4 - 1; 3 – 2 |
| Post-BD FEV_1_/FVC, % | 15.225 | 1.0029E-7 | 6.9988 | 2.6743E-7 | 2 - 1; 3 - 1; 4 – 1 |
| Pre-BD FVC, L | 14.838 | 1.4174E-7 | 6.8485 | 3.489E-7 | 2 - 1; 3 - 1; 4 – 1 |
| Age, years | 14.309 | 2.2859E-7 | 6.6409 | 5.2249E-7 | 1 - 2; 1 - 3; 1 - 4; 2 – 3 |
| Post-BD FEF_25%-75%_, % predicted value | 13.068 | 7.1969E-7 | 6.1429 | 1.5353E-6 | 2 - 1; 3 - 1; 4 – 1 |
| Serum total IgE, kU/L | 12.383 | 1.3771E-6 | 5.861 | 2.7541E-6 | 3 - 1; 1 - 4; 3 - 2; 3 – 4 |
| Post-BD FEV_1_, % predicted value | 12.018 | 1.955E-6 | 5.7088 | 3.68E-6 | 2 - 1; 3 - 1; 4 – 1 |
| Post-BD PEF, % predicted value | 11.548 | 3.0838E-6 | 5.5109 | 5.4823E-6 | 2 - 1; 3 - 1; 4 – 1 |
| Post-BD PEF, L | 11.427 | 3.4723E-6 | 5.4594 | 5.8481E-6 | 2 - 1; 3 - 1; 4 – 1 |
| Pre-BD FVC, % predicted | 8.8285 | 4.8507E-5 | 4.3142 | 7.7612E-5 | 2 - 1; 3 - 1; 4 – 1 |
| Post-BD FVC | 8.6553 | 5.8216E-5 | 4.235 | 8.8711E-5 | 2 - 1; 3 - 1; 4 – 1 |
| Serum specific IgE, kU/L | 7.0816 | 3.18E-4 | 3.4976 | 4.6254E-4 | 3 - 1; 1 - 4; 3 - 2; 3 – 4 |
| Blood eosinophil cell counts, % | 4.4221 | 0.0066249 | 2.1788 | 0.0088332 | 1 - 2; 1 - 3; 1 – 4 |
| Serum κ plus λ Ig free light chains, kU/L | 3.934 | 0.011819 | 1.9274 | 0.015129 | 1 - 2; 1 - 3; 1 – 4 |
| Serum λ Ig free light chains, kU/L | 3.6184 | 0.017235 | 1.7636 | 0.020463 | 1 - 2; 1 - 3; 1 – 4 |
| Serum κ Ig free light chains, kU/L | 3.6169 | 0.017266 | 1.7628 | 0.020463 | 1 - 2; 1 - 3; 1 – 4 |
| Pack-years | 3.0117 | 0.03579 | 1.4462 | 0.040903 | 1 - 3; 1 – 4 |

ANOVA with Tukey’s post-hoc test was used for between group comparisons. Parameters with more than 50% missing values were excluded. Group 1: severe persistent asthma; group 2: moderate persistent asthma; group 3: mild persistent asthma; group 4: healthy control.

Abbreviations: BD, bronchodilator; FEV_1,_ forced expiratory volume in 1 second; FVC, forced vital capacity; PEF, peak expiratory flow; FEV_1_/FVC%, FEV_1_ as percent of FVC; FEF_F25-75%_, forced expiratory flow at 25%-75% of FVC; IgE, immunoglobulin E.
