# Supplementary material for: Elevated serum polyclonal immunoglobulin free light chains in patients with severe asthma
Source: Front Pharmacol. 2023 Jun 16;14:1126535. doi: 10.3389/fphar.2023.1126535 (PMC10311563; doi:10.3389/fphar.2023.1126535)
Supplement: Supplementary file 6 [file Presentation1.pdf]

Supplementary file text

RESULTS

Correlations

Figure 5 shows the correlations of the study outcome as a heatmap. Figure 6 shows the correlation network. Serum κ and λ FLC concentrations were highly correlated (Pearson r = 0.85786, P = $1.6535E^{-22}$, FDR = $1.7637E^{-21}$) and correlated with age (Figure 5, Figure 7, Figure 8, Table S3, Table S4, Figure S4). A negative correlation was observed between serum κ and λ FLC concentrations and most pulmonary function testing outcomes (Figure 5, Figure 7, Figure 8, Table S3, Table S4, Figure S4).

In adults with persistent severe asthma, serum FLC were highly correlated with inflammatory outcomes reflecting T2 high, including peripheral blood eosinophil as percentage of total counts (serum κ FLC, Pearson r = 0.51297, P = $2.9678E^{-6}$, FDR = $2.3742E^{-5}$; serum λ FLC, Pearson r = 0.42297, P = $1.7377E^{-4}$, FDR = $9.2677E^{-4}$) and absolute values (serum κ FLC, Pearson r = 0.44853, P = $6.1284E^{-5}$, FDR = $3.2685E^{-4}$; serum λ FLC, Pearson r = 0.38204, P = $7.8261E^{-4}$, FDR = 0.0031304), with peripheral blood neutrophils as percentage of total counts (serum κ FLC, Pearson r = 0.30752, P = 0.0076929, FDR = 0.015386; serum λ FLC, Pearson r = 0.28735, P = 0.01305, FDR = 0.024565) and absolute values (serum κ FLC, Pearson r = 0.40151, P = $3.9176E^{-4}$, FDR = 0.001567; serum λ FLC, Pearson r = 0.39741, P = $4.5479E^{-4}$, FDR = 0.002079), and with serum CRP concentrations (serum κ FLC, Pearson r = 0.33473, P = 0.0035562, FDR = 0.008096; serum λ FLC, Pearson r = 0.37852, P = $8.8305E^{-4}$, FDR = 0.0031397) (Figure 7, Figure 8, Table S3, Table S4, Figure S4) (correlation table and p values correlation table supplementary files). By contrast, serum FLC concentrations were not correlated with other surrogate markers reflecting T2 high airway inflammation, including F_ENO (serum κ FLC, Pearson r = 0.1178, P = 0.31752, FDR = 0.37632; λ FLC, Pearson r = 0.16824, P = 0.15189, FDR = 0.18694), serum total IgE (serum κ

FLC, Pearson r = 0.05329, P = 0.65204, FDR = 0.69551; serum λ FLC, Pearson r = 0.064342, P = 0.58601, FDR = 0.62507) or specific IgE (serum κ FLC, Pearson r = 0.12854, P = 0.27506, FDR = 0.33854; serum λ FLC, Pearson r = 0.1333, P = 0.25753, FDR = 0.30522) (Table S3 and Table S4) (correlation table and p values correlation table supplementary files). Correlations of κ FLC or λ FLC serum concentrations with peripheral blood lymphocytes and monocytes are shown in Figure S5 and Figure S6, respectively. Linear regression analyses of κ FLC or λ FLC serum concentrations and peripheral blood lymphocytes and monocytes are shown in Figure S7 and Figure S8, respectively. Serum FLC concentrations were correlated with peripheral blood lymphocyte absolute values (κ FLC: Pearson r = 0.45, $P < 0.05$, n = 23 (Figure S5A); λ FLC: Pearson r = 0.53, $P < 0.05$, n = 23 (Figure 6A), but not with lymphocytes as percentage of total cell counts (Figure S5B and Figure S6B), monocyte absolute values (Figure S5C and Figure S6C) or monocytes as percentage of total cell count (Figure S5D and Figure S6D).

Figure legend

Figure S1. ROC curves showing classification of severe asthma individuals versus healthy control individuals (A) or severe asthma individuals versus mild asthma individuals (B) using measurement of serum κ free light chain (FLC) concentrations and classification of severe asthma individuals versus healthy control individuals (C) or severe asthma individuals versus mild asthma individuals (D) using measurement of serum λ FLC concentrations.

Figure S2. Pre-bronchodilator lung function test values in persistent severe asthma (dots), persistent moderate asthma (squares), persistent mild asthma (up-pointing triangles) and healthy control subjects (down-pointing triangles). Forced expiratory volume in one second (FEV_1) as absolute values (A), FEV_1 as percentage of predicted values (B), forced vital capacity (FVC) as

absolute values (C), FVC as percentage of predicted values (D), FEV_1/FVC ratio (E), peak expiratory flow (PEF) as absolute values (F), PEF as percentage of predicted values (G), forced expiratory flow at 25%-75% of FVC ($FEF_{25\%-75\%}$) as absolute values (H) and $FEF_{25\%-75\%}$ as percentage of predicted values (I). Mean values with SD are shown. $P < 0.05$ was considered significant.

Figure S3. Post-bronchodilator lung function test values in persistent severe asthma (dots), persistent moderate asthma (squares), persistent mild asthma (up-pointing triangles) and healthy control subjects (down-pointing triangles). Forced expiratory volume in one second (FEV_1) as absolute values (A), FEV_1 as percentage of predicted values (B), forced vital capacity (FVC) as absolute values (C), FVC as percentage of predicted values (D), FEV_1/FVC ratio (E), peak expiratory flow (PEF) as absolute values (F), PEF as percentage of predicted values (G), forced expiratory flow at 25%-75% of FVC ($FEF_{25\%-75\%}$) as absolute values (H) and $FEF_{25\%-75\%}$ as percentage of predicted values (I). Mean values with SD are shown. $P < 0.05$ was considered significant.

Figure S4. Top 25 study variables correlated with the sum of serum κ and λ free light chain (FLC) concentrations. Values of Pearson's correlation coefficients are shown.

Figure S5. Correlations of κ free light chain (FLC) serum concentrations with peripheral blood lymphocyte absolute values (A) ($r = 0.45$, $P < 0.05$, $n = 23$), lymphocytes as percentage of total cell counts (B) ($r = -0.01$, P = n.s, $n = 23$), monocyte absolute values (C) ($r = 0.32$, P = n.sn., $n = 23$), and monocytes as percentage of total cell counts (D) ($r = -0.20$, P = n.s., $n = 23$) in adults with persistent severe asthma. Values of Pearson's correlation coefficients are shown. $P < 0.05$ was considered significant. Abbreviation: n.s., not significant.

Figure S6. Correlations of λ free light chain (FLC) serum concentrations with peripheral blood lymphocyte absolute values (A) ($r = 0.53$, $P < 0.05$, $n = 23$), lymphocytes as percentage of total cell counts (B) ($r = 0.10$, P = n.s, $n = 23$), monocyte absolute values (C) ($r = 0.25$, P = n.sn., $n = 23$), and monocytes as percentage of total cell counts (D) ($r = -0.28$, P = n.s., $n = 23$) in adults with persistent severe asthma. Values of Pearson's correlation coefficients are shown. $P < 0.05$ was considered significant. Abbreviation: n.s., not significant.

Figure S7. Linear regression analyses of κ free light chain (FLC) serum concentrations and peripheral blood lymphocyte absolute values (A) ($R^2 = 0.20$, $P < 0.05$, $n = 23$) , lymphocytes as percentage of total cell counts (B) ($R^2 = 0.0001$, P = n.s, $n = 23$), monocyte absolute values (C) ($R^2 = 0.10$, P = n.s.., $n = 23$), and monocytes as percentage of total cell counts (D) ($R^2 = 0.04$, P = n.s., $n = 23$) in adults with persistent severe asthma. R^2 values are shown. $P < 0.05$ was considered significant. Abbreviation: n.s., not significant.

Figure S8. Linear regression analyses of λ free light chain (FLC) serum concentrations and peripheral blood lymphocyte absolute values (A) ($R^2 = 0.28$, $P < 0.01$, $n = 23$), lymphocytes as percentage of total cell counts (B) ($R^2 = 0.01$, P = n.s, $n = 23$), monocyte absolute values (C) ($R^2 = 0.06$, P = n.s.., $n = 23$), and monocytes as percentage of total cell counts (D) ($R^2 = 0.08$, P = n.s., $n = 23$) in adults with persistent severe asthma. R^2 values are shown. $P < 0.05$ was considered significant. Abbreviation: n.s., not significant.
