# Supplementary material for: Elevated serum polyclonal immunoglobulin free light chains in patients with severe asthma
Source: Front Pharmacol. 2023 Jun 16;14:1126535. doi: 10.3389/fphar.2023.1126535 (PMC10311563; doi:10.3389/fphar.2023.1126535)
Supplement: Supplementary file 10 [file Table2.docx]

**Table S2.** Serum Free Light Chain (FLC) concentrations ​​in females and males in each study group^§^

|  | Severe asthmatics | | P value | Steroid-treated moderate asthmatics | | P value | Steroid-naïve mild asthmatics | | P value | Healthy control subjects | | P value |
| --- | --- | --- | --- | --- | --- | --- | --- | --- | --- | --- | --- | --- |
|  | Females | Males |  | Females | Males |  | Females | Males |  | Females | Males |  |
| n | 15 | 9 |  | 7 | 8 |  | 6 | 9 |  | 12 | 8 |  |
| κ FLC, mg/l | 15.3±5.1^,^ | 17.2±6.2 | 0.39 | 13.5±4.8 | 12.2±2.5 | 0.52 | 13.6±3.8 | 11.1±3.5 | 0.22 | 11.6±3.9 | 13.2±2.9 | 0.33 |
| λ FLC, mg/l | 21.0±8.9 | 25.3±11.0 | 0.31 | 18.1±8.5 | 16.1±3.0 | 0.54 | 19.9±6.0 | 14.3±5.7 | 0.09 | 16.9±4.7 | 16.7±3.8 | 0.89 |
| κ/λ ratio, % | 0.8±0.1 | 0.7±0.2 | 0.51 | 0.8±0.3 | 0.8±0.1 | 0.77 | 0.7±0.3 | 0.8±0.1 | 0.07 | 0.7±0.2 | 0.8±0.1 | 0.18 |
| κ + λ, mg/l | 36.2±13.7 | 42.5±16.7 | 0.33 | 31.6±12.4 | 28.3±5.2 | 0.50 | 33.5±9.7 | 25.3±9.0 | 0.12 | 28.5±7.8 | 29.9±6.5 | 0.69 |

Abbreviations: FLC, free light chain.

^§^Data are expressed as mean ± SD. Data were normally distributed after log transformation. Frequency of distribution was assessed with the D'Agostino-Pearson omnibus normality test. Unpaired t test was used for between group comparison. Significance was defined as a value of P < 0.05.
