# Supplementary material for: Elevated serum polyclonal immunoglobulin free light chains in patients with severe asthma
Source: Front Pharmacol. 2023 Jun 16;14:1126535. doi: 10.3389/fphar.2023.1126535 (PMC10311563; doi:10.3389/fphar.2023.1126535)
Supplement: Supplementary file 11 [file Table3.docx]

**Table S3.** Correlations of serum κ IgE free light chain (FLC) concentrations

|  | Pearson r correlation coefficient | T stat | P value | FDR |
| --- | --- | --- | --- | --- |
| serum κ plus λ Ig FLC concentrations, kU/L | 0.94355 | 24.172 | 2.7855E-36 | 4.4568E-35 |
| serum λ Ig FLC concentrations, kU/L | 0.85786 | 14.165 | 1.6535E-22 | 1.7637E-21 |
| blood eosinophil cell counts, % | 0.51297 | 5.0707 | 2.9678E-6 | 2.3742E-5 |
| age | 0.47785 | 4.6157 | 1.6667E-5 | 1.0667E-4 |
| blood eosinophil cells, absolute values | 0.44853 | 4.2582 | 6.1284E-5 | 3.2685E-4 |
| post-BD FEF_25%-75%_, L/s | -0.42855 | -4.0246 | 1.3938E-4 | 6.3716E-4 |
| blood neutrophil cell counts, absolute values | 0.40151 | 3.72 | 3.9176E-4 | 0.001567 |
| post-BD FEF_25%-75%_, % predicted values | -0.355 | -3.2222 | 0.0019117 | 0.0067973 |
| pre-BD FEF_25%-75%_, L/s | -0.3506 | -3.1765 | 0.0021954 | 0.0070252 |
| post-BD FEV_1_, L | -0.34576 | -3.1267 | 0.0025498 | 0.0074176 |
| pre-BD FEV_1_/FVC, % | -0.3364 | -3.0312 | 0.0033839 | 0.008096 |
| serum CRP | 0.33473 | 3.0142 | 0.0035562 | 0.008096 |
| pre-BD FEV_1,_ L | -0.33359 | -3.0026 | 0.0036785 | 0.008096 |
| post-BD FEV_1_/FVC, % | -0.33253 | -2.9919 | 0.003795 | 0.008096 |
| blood neutrophil cell counts, % | 0.30752 | 2.7423 | 0.0076929 | 0.015386 |
| post-BD FVC, L | -0.28578 | -2.5304 | 0.013579 | 0.025561 |
| pre-BD FVC, L | -0.2711 | -2.3899 | 0.019473 | 0.034618 |
| pre-BD FEF_25%-75%,_ % predicted values | -0.23607 | -2.0614 | 0.042874 | 0.072209 |
| pre-BD PEF, L | -0.23025 | -2.0077 | 0.048426 | 0.074319 |
| post-BD PEF, L/s | -0.22991 | -2.0046 | 0.048772 | 0.074319 |
| post-BD FEV_1_, % predicted values | -0.2124 | -1.8443 | 0.069247 | 0.09828 |
| pre-BD FEV_1_, % predicted values | -0.21137 | -1.835 | 0.070639 | 0.09828 |
| pre-BD PEF, % predicted values | -0.16961 | -1.4603 | 0.14855 | 0.19807 |
| post-BD PEF, % predicted values | -0.13217 | -1.1314 | 0.26162 | 0.33488 |
| serum specific IgE concentrations, kU/L | 0.12854 | 1.0998 | 0.27506 | 0.33854 |
| F_E_NO, ppb | 0.1178 | 1.0066 | 0.31752 | 0.37632 |
| pre-BD FVC, % predicted values | -0.066344 | -0.56419 | 0.57438 | 0.65643 |
| post-BD FVC, % predicted values | -0.054281 | -0.46127 | 0.646 | 0.69551 |
| serum total IgE concentrations, kU/L | 0.05329 | 0.45282 | 0.65204 | 0.69551 |
| pack/years | 0.020089 | 0.1705 | 0.8651 | 0.893 |
| κ/λ Ig FLC ratio | -0.005105 | -0.043318 | 0.96557 | 0.96557 |

Abbreviations: BD, bronchodilator; CRP, C reactive protein; FDR, false discovery rate; F_E_NO, fractional exhaled nitric oxide; FEV_1,_ forced expiratory volume in 1 second; FEF_F25-75%_, forced expiratory flow at 25%-75% of FVC; FEV_1_/FVC%, FEV_1_ as percent of FVC; FVC, forced vital capacity; IgE, immunoglobulin E; PEF, peak expiratory flow.
