# Supplementary material for: Elevated serum polyclonal immunoglobulin free light chains in patients with severe asthma
Source: Front Pharmacol. 2023 Jun 16;14:1126535. doi: 10.3389/fphar.2023.1126535 (PMC10311563; doi:10.3389/fphar.2023.1126535)
Supplement: Supplementary file 12 [file Table4.docx]

**Table S4**. Correlations of serum λ Ig free light chain (FLC) concentrations

|  | Pearson r correlation coefficient | T stat | P value | FDR |
| --- | --- | --- | --- | --- |
| serum κ plus λ Ig FLC concentrations, kU/L | 0.97962 | 41.383 | 6.1578E-52 | 9.8524E-51 |
| serum κ Ig FLC concentrations, kU/L | 0.85786 | 14.165 | 1.6535E-22 | 1.7637E-21 |
| κ/λ Ig FLC ratio | -0.48025 | -4.6458 | 1.4902E-5 | 1.1922E-4 |
| Age | 0.42585 | 3.9937 | 1.5513E-4 | 9.2677E-4 |
| blood eosinophil cell counts, % | 0.42297 | 3.9607 | 1.7377E-4 | 9.2677E-4 |
| blood neutrophil cell counts, absolute values | 0.39741 | 3.6748 | 4.5479E-4 | 0.002079 |
| blood eosinophil cells, absolute values | 0.38204 | 3.5078 | 7.8261E-4 | 0.0031304 |
| serum CRP | 0.37852 | 3.47 | 8.8305E-4 | 0.0031397 |
| post-BD FEF_25%-75%_, L/s | -0.33702 | -3.0374 | 0.0033221 | 0.0096319 |
| pre-BD FEV_1_, L | -0.33536 | -3.0206 | 0.0034903 | 0.0096319 |
| pre-BD FEV_1_/FVC, % | -0.33421 | -3.0089 | 0.003612 | 0.0096319 |
| pre-BD FEF_25%-75%_, L/s | -0.32772 | -2.9434 | 0.0043669 | 0.010406 |
| post-BD FEV_1_, L | -0.32628 | -2.9289 | 0.0045526 | 0.010406 |
| post-BD FEV_1_/FVC, % | -0.29099 | -2.5809 | 0.011893 | 0.024284 |
| pre-BD FVC, L | -0.29019 | -2.573 | 0.012142 | 0.024284 |
| blood neutrophil cell counts, % | 0.28735 | 2.5456 | 0.01305 | 0.024565 |
| post-BD FVC, L | -0.2767 | -2.4433 | 0.017006 | 0.030233 |
| post-BD FEF_25%-75%,_ % predicted values | -0.25921 | -2.2773 | 0.025741 | 0.043353 |
| pre-BD PEF, L | -0.25245 | -2.2138 | 0.030009 | 0.048014 |
| pre-BD FEF_25%-75%,_ % predicted values | -0.23935 | -2.0918 | 0.039988 | 0.060935 |
| pre-BD FEV_1_, % predicted values | -0.22547 | -1.9637 | 0.053426 | 0.077711 |
| post-BD PEF, L/s | -0.22147 | -1.9271 | 0.057911 | 0.080572 |
| post-BD FEV_1_, % predicted values | -0.19577 | -1.694 | 0.094595 | 0.12613 |
| pre-BD PEF, % predicted values | -0.19011 | -1.6431 | 0.10472 | 0.13404 |
| F_E_NO, ppb | 0.16824 | 1.4482 | 0.15189 | 0.18694 |
| serum specific IgE concentrations, kU/L | 0.1333 | 1.1413 | 0.25753 | 0.30522 |
| post-BD PEF, % predicted values | -0.11528 | -0.98479 | 0.32803 | 0.37489 |
| pre-BD FVC, % predicted values | -0.10053 | -0.8574 | 0.39407 | 0.43484 |
| serum total IgE concentrations, kU/L | 0.064342 | 0.54709 | 0.58601 | 0.62507 |
| post-BD FVC, % predicted values | -0.044583 | -0.37867 | 0.70604 | 0.72882 |
| pack/years | 0.0067583 | 0.057348 | 0.95443 | 0.95443 |

Abbreviations: BD, bronchodilator; CRP, C reactive protein; FDR, false discovery rate; F_E_NO, fractional exhaled nitric oxide; FEV_1,_ forced expiratory volume in 1 second; FEF_F25-75%_, forced expiratory flow at 25%-75% of FVC; FEV_1_/FVC%, FEV_1_ as percent of FVC; FVC, forced vital capacity; IgE, immunoglobulin E; PEF, peak expiratory flow.
